# Functional, Antigen-Specific Stem Cell Memory (T_SCM_) CD4^+^ T Cells Are Induced by Human *Mycobacterium tuberculosis* Infection

**DOI:** 10.3389/fimmu.2018.00324

**Published:** 2018-03-01

**Authors:** Cheleka A. M. Mpande, One B. Dintwe, Munyaradzi Musvosvi, Simbarashe Mabwe, Nicole Bilek, Mark Hatherill, Elisa Nemes, Thomas J. Scriba, The SATVI Clinical Immunology Team, Cynthia Ontong

**Affiliations:** ^1^South African Tuberculosis Vaccine Initiative, Institute of Infectious Disease and Molecular Medicine, Division of Immunology, Department of Pathology, University of Cape Town, Cape Town, South Africa

**Keywords:** T_SCM_, *Mycobacterium tuberculosis*, memory T cells, QuantiFERON conversion, LTBI

## Abstract

**Background:**

Maintenance of long-lasting immunity is thought to depend on stem cell memory T cells (T_SCM_), which have superior self-renewing capacity, longevity and proliferative potential compared with central memory (T_CM_) or effector (T_EFF_) T cells. Our knowledge of T_SCM_ derives primarily from studies of virus-specific CD8^+^ T_SCM_. We aimed to determine if infection with *Mycobacterium tuberculosis* (*M. tb*), the etiological agent of tuberculosis, generates antigen-specific CD4^+^ T_SCM_ and to characterize their functional ontology.

**Methods:**

We studied T cell responses to natural *M. tb* infection in a longitudinal adolescent cohort of recent QuantiFERON-TB Gold (QFT) converters and three cross-sectional QFT^+^ adult cohorts; and to bacillus Calmette–Guerin (BCG) vaccination in infants. *M. tb* and/or BCG-specific CD4 T cells were detected by flow cytometry using major histocompatibility complex class II tetramers bearing Ag85, CFP-10, or ESAT-6 peptides, or by intracellular cytokine staining. Transcriptomic analyses of *M. tb*-specific tetramer^+^ CD4^+^ T_SCM_ (CD45RA^+^ CCR7^+^ CD27^+^) were performed by microfluidic qRT-PCR, and functional and phenotypic characteristics were confirmed by measuring expression of chemokine receptors, cytotoxic molecules and cytokines using flow cytometry.

**Results:**

*M. tb*-specific T_SCM_ were not detected in QFT-negative persons. After QFT conversion frequencies of T_SCM_ increased to measurable levels and remained detectable thereafter, suggesting that primary *M. tb* infection induces T_SCM_ cells. Gene expression (GE) profiling of tetramer^+^ T_SCM_ showed that these cells were distinct from bulk CD4^+^ naïve T cells (T_N_) and shared features of bulk T_SCM_ and *M. tb*-specific tetramer^+^ T_CM_ and T_EFF_ cells. These T_SCM_ were predominantly CD95^+^ and CXCR3^+^, markers typical of CD8^+^ T_SCM_. Tetramer^+^ T_SCM_ expressed significantly higher protein levels of CCR5, CCR6, CXCR3, granzyme A, granzyme K, and granulysin than bulk T_N_ and T_SCM_ cells. *M. tb*-specific T_SCM_ were also functional, producing IL-2, IFN-γ, and TNF-α upon antigen stimulation, and their frequencies correlated positively with long-term BCG-specific CD4^+^ T cell proliferative potential after infant vaccination.

**Conclusion:**

Human infection with *M. tb* induced distinct, antigen-specific CD4^+^ T_SCM_ cells endowed with effector functions, including expression of cytotoxic molecules and Th1 cytokines, and displayed chemokine receptor profiles consistent with memory Th1/17 cells. Induction of CD4^+^ T_SCM_ should be considered for vaccination approaches that aim to generate long-lived memory T cells against *M. tb*.

## Introduction

Memory T cells have been classified into subsets, according to their phenotypes, functions and homing potential (1). Antigen-specific central memory T cells (T_CM_, CD45RA^−^ CCR7^+^) have been considered the main mediators of maintenance and expansion of T cell immunity, following secondary antigen exposure, due to their ability to differentiate into effector T cells (T_EFF_, CD45RA^−^ CCR7^−^), as well as their increased proliferative capacity and longevity compared with T_EFF_ cells. This ontology of memory T cells has recently been revised to include a new subset termed stem cell memory T cells (T_SCM_). T_SCM_ typically express CD45RA, CCR7, and CD27, and thus phenotypically resemble naïve T cells, but their co-expression of memory markers, such as CD95 and CXCR3, distinguish them from naïve T cells (2, 3). Functional characterization of antigen-specific T_SCM_ has predominantly foc-used on viral, parasitic, and tumor-specific CD8 T cells, identified by major histocompatibility complex (MHC) class I tetramers, or by non-specific and/or antigen specific stimulation (2–6). These studies showed that T_SCM_ characteristically possess excellent self-renewing capacity, longevity, proliferative capacity, relative to T_CM_ and T_EFF_. Importantly, T_SCM_ can also differentiate into T_CM_ and/or T_EFF_ cells (2). CD8^+^ T_SCM_ are preferentially enriched in the absence of antigen (3), non-chronic states of infection (6) and among long-lived T cells induced by vaccination (7). CD8^+^ T_SCM_ were also essential for the re-establishment of Ag-specific memory responses after T cell depletion in cancer patients (8, 9). As a result, T_SCM_ T cells are considered as a potential target for vaccination against infectious diseases and T cell therapy for autoimmunity, that aim at inducing and maintaining long-lasting T cell immunity capable of replenishing all T cell memory subsets.

Animal models of tuberculosis (TB) and human studies show that CD4 T cells, and especially those that have differentiated into antigen-specific Th1 cells, are necessary for immunological control of the intracellular bacterium, *Mycobacterium tuberculosis* (*M. tb*) [reviewed in Ref. (10)]. Newborn vaccination against TB with the bacillus Calmette–Guerin (BCG) vaccine is efficacious against severe forms of TB, such as milliary and meningitic TB, in young children (11, 12). Efficacy of BCG against pulmonary disease after childhood is variable and mostly poor (13, 14). As a consequence, it has been proposed that waning of mycobacteria-specific T cell memory responses and insufficient maintenance of protective CD4^+^ T cells may underlie the limited durability of BCG-induced protection (15, 16).

Newborn BCG vaccination induces antigen-specific T_CM_ and T_EFF_ CD4^+^ T cell responses (17), but the role of CD4^+^ T_SCM_ cells in immune responses induced by vaccination against TB, or by natural *M. tb* infection, in humans has not been explored. In fact, there is very limited knowledge about the functional capacity and persistence of CD4^+^ T_SCM_ that are specific for bacterial antigens. We and others have reported that a considerable proportion of cytokine-expressing or tetramer^+^ mycobacteria-specific CD4^+^ T cells, in humans, displayed a memory phenotype characteristic of naïve T cells (CD45RA^+^ CCR7^+^), and termed them naïve-like CD4^+^ T cells (17–22). In a clinical trial that tested boosting of mycobacteria-specific responses with the TB vaccine candidate, MVA85A, low but detectable Ag85A-specific CD45RA^+^ CCR7^+^ CD27^+^ naive-like CD4^+^ T cell responses were observed before MVA85A vaccination and frequencies of these cells remained unchanged after vaccination (23). In addition, a murine study demonstrated that BCG-induced naïve-like (CD44^lo^ CD62L^hi^) memory cells played a role in the control of *M. tb* infection, where these cells were capable of replenishing effector (CD44^hi^ CD62L^lo^) T cells with superior functional activity and protective potential against *M. tb* infection, compared with those originating from effector T cells (24). The characteristics of such mycobacteria-specific naïve-like CD4^+^ T cells are thus consistent with those of CD4^+^ T_SCM_ cells.

We hypothesized that *M. tb*-specific CD4^+^ T_SCM_ are induced by primary infection with *M. tb* in humans and aimed to determine the kinetics of their generation and to characterize gene expression (GE), homing potential and functional profiles of mycobacteria-specific CD4^+^ T_SCM_. Phenotypic and functional properties of *M. tb*-specific T_SCM_ were compared with those of *M. tb*-specific T_CM_ and T_EFF_, to determine their ontology. Our findings contribute to the current knowledge of the *M. tb*-specific T cell memory repertoire and highlight the need for a better understanding of CD4^+^ T_SCM_ cells in natural *M. tb* infection, TB disease and vaccine-induced immune responses.

## Materials and Methods

### Study Participants

Consent forms and study protocols were approved by the Human Research Ethics Committee of the University of Cape Town (UCT HREC 126/2006, 045/2008, 179/2011, 013/2012, 753/2014). Healthy adults with a positive QuantiFERON Gold In-Tube (QFT) test (IFN-γ > 0.35 IU/mL) were recruited from the community living in the Worcester region of Western Cape, South Africa. All participants provided written informed consent. Inclusion criteria included age above 18 years, QFT-positive, HIV-seronegative, and no prior (*self-reported*) or current signs or symptoms suggestive of TB disease.

We also retrieved cryopreserved peripheral blood mononuclear cells (PBMC) from a subset of 12- to 18-year-old adolescent participants with evidence of newly acquired *M. tb* infection, from the longitudinal Adolescent Cohort Study (25). Parents or legal guardians of adolescents provided written informed con-sent and adolescents provided written informed assent. New *M. tb* infection was defined as a negative Tuberculin Skin Test (TST) (induration = 0 mm) and negative QFT test (IFN-γ < 0.35 IU/mL), followed by at least three positive QFT tests 6, 12, and 18 months later and a positive TST (induration > 10 mm) at 12 months.

We also performed new analyses of existing immune response data from healthy HIV-exposed but uninfected infant participants of a recently published clinical trial [see Ref. (26) for details; http://ClinicalTrials.gov NCT01650389]. Participants of this trial received either MVA85A vaccination or placebo (Candin^®^, AllerMed) at birth and, if confirmed HIV-PCR negative, BCG vaccination at 8 weeks of age, after which they were followed up for 44 weeks. Analyses reported here include only infants who received placebo at birth.

### Blood Processing and Stimulation for Intracellular Cytokine Staining Assay

Peripheral blood mononuclear cells from adults were isolated by density gradient centrifugation (Ficoll histopaque, Lonza) from blood collected in sodium (Na)-heparin tubes (Greiner Bio-one) or heparinized blood bags. PBMC were analyzed fresh or cryopreserved in RPMI 1640 media (RPMI, Lonza) with 10% v/v dimethyl sulfoxide (DMSO, Sigma-Aldrich) and 45% v/v fetal bovine serum (Biochrom).

Whole blood intracellular cytokine staining (WB-ICS) assays were performed as described previously (26–28). Briefly, 1 mL whole blood was either left unstimulated (negative control) or stimulated with phytohemagglutinin (at 10 μg/mL, positive control), peptide pools of Ag85B, ESAT-6, or CFP-10 (all 15mer peptides, overlapping by 10 aa at 2 μg/mL, GenScript) or BCG (≈1.2 × 10^6^ CFU/mL, Statens Serum Institut) for 12 h or 7 days (BCG only, used at 1 × 10^5^ CFU/mL). Thereafter, red cells were lysed and white cells fixed using FACS-Lysing solution (BD Biosciences), before cryopreservation in 10% DMSO in fetal calf serum.

### Flow Cytometry

Multiparameter flow cytometry panels were designed (Table S1 in Supplementary Material) to sort memory subsets as bulk or *M. tb*-tetramer^+^ CD4^+^ T cells (panel 1, Figure S1A in Supplemen-tary Material), measure *M. tb*-specific CD4^+^ T cell kinetics after *M. tb* infection (panel 2, Figure S1B in Supplementary Mater-ial), determine *M. tb*-specific CD4^+^ T cell chemokine receptor (panel 3, Figure S1C in Supplementary Material), cytotoxic molecule (panel 4, Figure S1D in Supplementary Material), and cytokine [panel 5, Figure S6A in Supplementary Material, see Ref. (28)] expression profiles. In addition, BCG vaccine-induced cytokine expression profiles [panel 6, see Ref. (26)] and proliferative capacity [panel 7, see Ref. (26)] of mycobacteria-specific CD4^+^ T cells were measured.

### MHC Class II Tetramers

Major histocompatibility complex class II tetramers conjugated to PE and/or APC were kindly provided by the National Institutes of Health (NIH) tetramer core facility. To exclude detection of non-specific MHC class II tetramer binding to B cells, CD8 T cells, monocytes, and dead cells, we gated CD4^+^ T cells on CD8^−^, CD14^−^, CD19^−^, live cells by including antibodies conjugated to the same fluorochrome to these markers (collectively termed a dump channel) (Figure S1A–D in Supplementary Material). *M. tb*-tetramer^+^ CD4^+^ T cells were detected using MHC class II tetramers conjugated to the following mycobacterial peptides: Ag85 [DPB1*04:01-Ag85B_128–144_ (GKAGCQTYKWETFLTSE), DRB1*03:01-Ag85A_56–75_ (VPSPSMGRDIKVQFQSGGAN)], CFP-10 [DRB1*04:01-CFP-10_71–85_ (EISTNIRQAGVQYSR), DRB5*01:01-CFP-10_51–65_ (AQAAVVRFQEAANKQ)], and ESAT-6 [DQB1*06:02-ESAT6_31–45_ (EGKQSLTKLAAAWGG)]. Non-specific tetramer staining was detected using MHC class II tetramers conjugated to human CLIP self-peptide, [DPB1*04:01-CLIP_81–101_ (PVSKMRMATPLLMQA), DQB1*06:02-CLIP_81–101_, DRB1*03:01-CLIP_81–101_, DRB1*04:01-CLIP_81–101_, and DRB5*01:01-CLIP_81–101_].

### Flow Cytometry Staining Protocol for Sorting (Flow Cytometry Panel 1)

Cryopreserved PBMC were thawed into medium containing DNAse (50 IU/mL, Sigma-Aldrich), washed and stained with Violet or Aqua LIVE/DEAD Fixable Dead Cell Stain (Thermo-Fisher Scientific). Cells were then stained with anti-CCR7 anti-body at 37°C for 20 min, washed and then stained with 2 µg/mL MHC class II-CFP10 tetramers (DRB1*04:01-CFP-10_71–85_, DRB5*01:01-CFP-10_51–65_) at room temperature (RT) for 1 h. Cells were washed and stained with surface marker antibodies, according to Table S1 in Supplementary Material, for 40 min at RT in a total volume of 100 µL. Samples were acquired and sorted on a BD Bioscience FACS Aria I sorter using FACS DIVA software (Version 6).

### Flow Cytometry Staining Protocol for PBMC (Panels 2–4)

#### Chemokine Receptor Staining

Cryopreserved or fresh PBMC were stained with antibodies to chemokine receptors (Table S1 in Supplementary Material) for 30 min at 37°C.

#### MHC Class II Tetramers Staining

During optimization experiments we noted that MHC class II tetramer staining of some samples yielded artifactual labeling which appeared to be fluorochrome-dependent. As a consequence, when necessary, PBMC were stained with two preparations of the identical tetramer, one conjugated to PE and the other to APC. This identified PE and APC double-positive cells, allowing rigorous identification of tetramer^+^ CD4^+^ T cells. Tetramer staining was performed using mycobacteria-specific tetramers at a concentration of 2 µg/mL per tetramer preparations for 1 h at RT.

#### Phenotypic Marker Staining

Peripheral blood mononuclear cells were stained with fluorescently labeled antibodies to phenotypic markers and viability dye, according to Table S1 in Supplementary Material, for 30 min at RT, before washing and fixation with 1% paraformaldehyde (Kimix).

#### Intracellular Cytotoxic Molecule Staining

To stain for cytotoxic molecules, PBMC were fixed and permeabilized using the BD CytoxFix/Perm (BD Biosciences) according to the manufacturer’s protocol. Permeabilized PBMC were then stained with fluorescently labeled antibodies to cytotoxic molecules, according to Table S1 in Supplementary Material, for 30 min at RT. This was followed by washing and fixation. Samples were acquired on a BD Bioscience LSRFortessa using FACS DIVA software (Version 8).

### Flow Cytometry Staining Protocol for Whole Blood Intracellular Cytokine (WB-ICS) Assay (Panels 5–7)

Cryopreserved, fixed cells from the whole blood stimulation were thawed, permeabilized using Perm/Wash Solution (BD Biosciences), washed and stained with anti-CCR7 at 37°C for 20 min, followed by addition of the remaining antibodies, and staining on ice for 40 min.

### High Throughput Microfluidic RT-qPCR on Sorted T Cells

Thirty cells (MHC class II tetramer^+^ or bulk CD4^+^ T cells from each memory subset) were sorted into PCR tubes containing 5 µL CellsDirect 2× reaction mix, 0.5 µL SuperScript™ III RT/Platinum^®^ Taq mix, 2.5 µL of pooled TaqMan GE primer-probe assays (at a concentration of 0.2× per assay), and 1 µL TE buffer (10 mM Tris, pH 8.0, and 0.1 mM EDTA). Selection of TaqMan assays was based on published literature of transcriptional profiles of T cell memory subsets (Table S2 in Supplementary Material). The amplification efficiency of each TaqMan GE assay was assessed as previously described (29) and found to be close to 100% (±10%, data not shown). Specific transcript amplification (STA) was performed using the following thermal profile: 20 min at 50°C to lyse cells and perform cDNA synthesis, followed by 2 min at 95°C, then 18 cycles of 95°C for 15 s and 60°C for 4 min. STA-cDNA samples were diluted fivefold and loaded onto a 96.96 Dynamic Array chip (Fluidigm) with TaqMan GE assays for qPCR using a BioMark HD System (Fluidigm) according to the manufacturer’s protocol.

### Data Analysis

Flow cytometry data was analyzed using FlowJo (Tree Star) versions v9.7 to v10.1r.1, Pestle version 1.8, and SPICE version 5.2–5.3 (30).

Threshold cycle (Ct) values were determined by the BioMark Real-time PCR Analysis software using linear derivative background correction and an amplification curve quality threshold of 0.65. We excluded data from sorted CD4^+^ T cells with undetectable levels (i.e., Ct = 40) of *CD4* and/or *B2M* from our analyses. For ease of interpretation, Ct values were transformed to Et values (40 − Ct), because a higher Et value indicates higher mRNA levels. Relative mRNA transcript levels (delta Et) in tetramer^+^ CD4^+^ T cells were derived by subtracting the B2M Et value from the Et values of the genes of interest, and average delta Et values were calculated from duplicate STA-cDNA samples.

The lower detection limit for tetramer^+^ CD4 T cells measured by flow cytometry panels 2–4 (0.0021% of CD4^+^ T cells) was calculated as the median frequency of negative control tetramer^+^ CD4 T cells plus the 95% confidence interval of the median absolute deviation of negative control tetramer proportions. In addition, frequencies of tetramer^+^ CD4^+^ T cells had to be at least fivefold higher than frequencies of the corresponding control tetramer^+^ CD4^+^ T cells. Chemokine and cytotoxic molecule expression profiles were assessed for cell subsets comprising 20 or more cells (e.g., tetramer^+^ T_CM_ or T_EFF_ cells).

Antigen-specific cytokine^+^ cells expressing a T_SCM_ (CD45RA^+^ CCR7^+^) phenotype detected in the WB-ICS assay (panel 5) were typically very infrequent, and in some donors less than 10 cells of this subset were detected per sample upon background subtraction. As a result, we did not perform in-depth cytokine co-expression or Boolean analyses, and we report frequencies of CD4^+^ T_SCM_ cells that express IFN-γ, TNF-α, and IL-2 in unstimulated and antigen-stimulated samples.

Statistical analyses were performed using GraphPad Prism v6/7, R version 3.0.1 or SPICE version 5.2–5.3 (30). Specific statistical analyses are clearly defined where applicable. Differences in mRNA transcript expression between CD4^+^ T cell populations were computed using the Kruskal–Wallis or Mann–Whitney tests at a *p*-value threshold of 0.05 and a false discovery rate (FDR) threshold of 0.05 [Benjamini–Hochberg method (31)]. Principal component analysis (PCA) plots and heat maps were generated using the prcomp and heatmap.2 functions in R. Differences in protein expression were computed using Kruskal–Wallis or Mann–Whitney tests. The Bonferroni and Benjamini–Hochberg (FDR < 0.05) methods were used to correct for multiple comp-arisons for up to four comparisons or more than four comparisons, respectively. Differences in pie charts depicting chemokine receptor and cytotoxic molecule co-expression profiles were calculated using non-parametric permutation test comparing the overall distribution between subset proportions using SPICE (30).

## Results

### CD45RA^+^ CCR7^+^ CD27^+^*M. tb*-Tetramer^+^ CD4 T Cells Are Not Naïve CD4 T Cells

We previously observed mycobacteria-specific CD4^+^ T cells that expressed a CD45RA^+^ CCR7^+^ naïve phenotype but exhibited features not consistent with T_N_, which we termed “naïve-like” CD4^+^ T cells. In four studies, we showed that these “naïve-like” CD4^+^ T cells expressed Th1 cytokines (17–20), functions characteristic of antigen-experienced T cells. Also, in another study (23), we detected “naïve-like” CD4^+^ T cells by MHC class II tetramer staining at frequencies that exceeded those typical of T_N_, at 1–5 cells/million CD4 T cells (32–34). We hypothesized that such mycobacteria-specific naïve-like CD4^+^ T cells are T_SCM_ cells and performed GE profiling of sorted CFP10-specific tetramer^+^ CD4 T cells that displayed such a naive memory phenotype (CD45RA^+^ CCR7^+^ CD27^+^, naïve-like memory cells, T_NLM_) from seven healthy, *M. tb*-infected donors with HLA alleles that corresponded to our tetramer reagents. Refer to Supplementary Information for the rationale for using MHC class II tetramers. We also sorted and profiled GE of tetramer^+^ T_CM_ (CD45RA^−^ CCR7^+^ CD27^+^) and T_EFF_ (CD45RA^−^ CCR7^−^) CD4 T cells as well as bulk naïve, T_SCM_, T_CM_ and T_EFF_ CD4 T cells (Figure S1A in Supplementary Material). Because less than 50% of the tetramer^+^ T_NLM_ CD4 T cells expressed CD95, as also shown previously (23), we focused on CD95- tetramer^+^ T_NLM_ cells. One participant did not have detectable tetramer^+^ T_NLM_ CD4 T cells.

**Figure 1:**
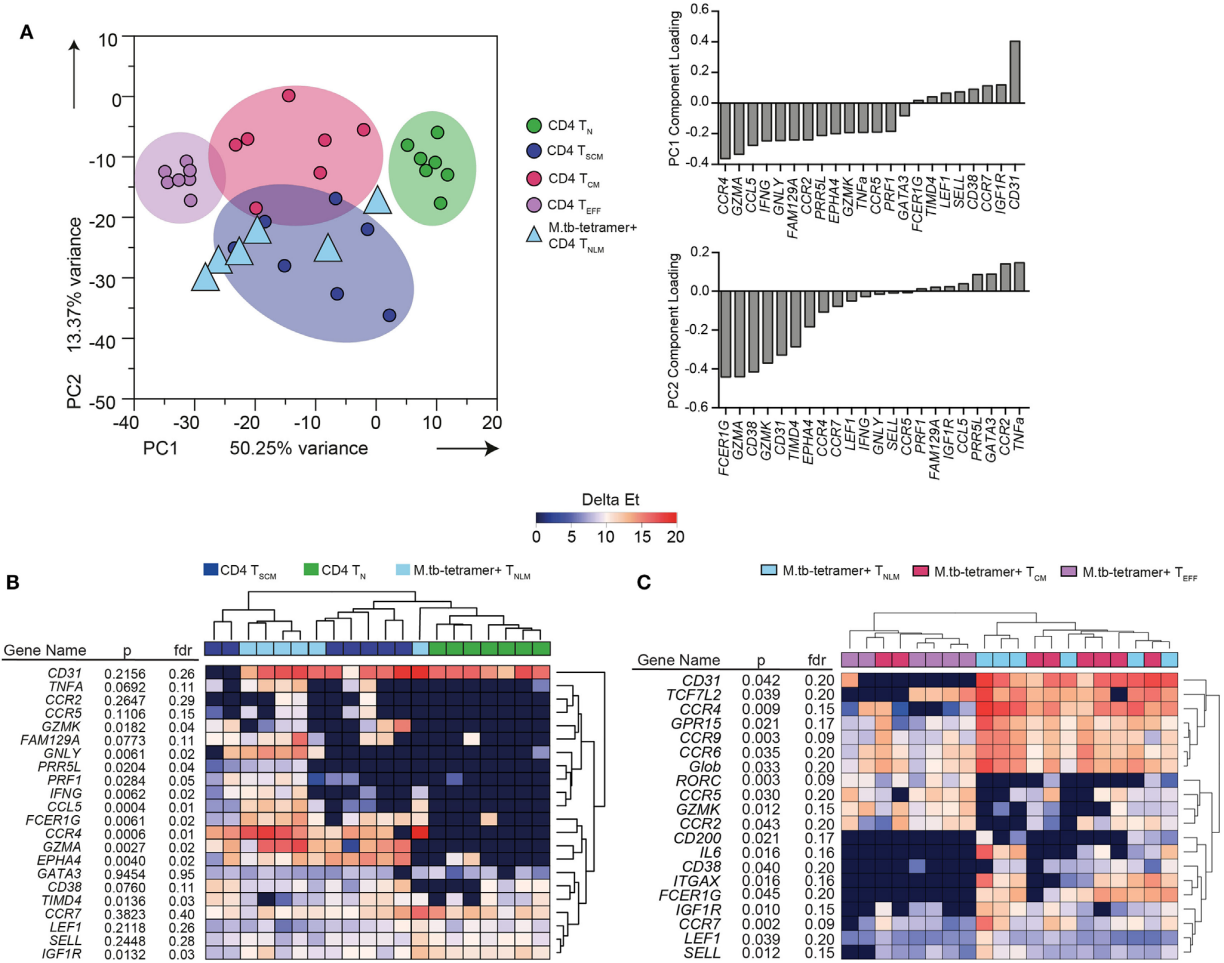
Transcriptional profile of *Mycobacterium tuberculosis* (*M. tb*)-specific T_SCM_. Expression of 96 genes was measured by microfluidic qRT-PCR on bulk or *M. tb*-tetramer^+^ CD4^+^ T cells expressing a naïve (T_N_, CD45RA^+^ CCR7^+^ CD27^+^ CD95^−^, labeled as naïve-like memory T_NLM_ for *M. tb*-tetramer^+^), T_SCM_ (CD45RA^+^ CCR7^+^ CD27^+^ CD95^+^, bulk CD4^+^ cells only), T_CM_ (CD45RA^−^ CCR7^+^ CD27^+^), or T_EFF_ (CD45RA^−^ CCR7^−^) phenotype, sorted from *M. tb*-infected adults (*n* = 7). Analyses shown in this figure were built on transcripts (22 mRNA) that were differentially expressed [Kruskal–Wallis *H* test *p* < 0.05 and false discovery rate (FDR) < 0.05] between bulk CD4^+^ T_N_, T_SCM_, T_CM_, and T_EFF_ cells. **(A)** Principal component analysis (PCA) of sorted bulk T_N_ (green), T_SCM_ (indigo), T_CM_ (magenta), and T_EFF_ (purple). PCA loadings—PC1 and PC2—defined on bulk CD4^+^ subsets were applied to *M. tb*-tetramer^+^ T_NLM_ (light blue triangles) CD4^+^ T cells. **(B)** Unsupervised heat map of 22 transcripts differentially expressed between bulk T_N_, T_SCM_, T_CM_, and T_EFF_ subsets. Bulk T_N_, T_SCM_, and *M. tb*-tetramer^+^ T_NLM_ represented as green, indigo, and light blue, respectively. Gene expression (GE) is reported as delta Et values [40 − threshold cycle (Ct) values]. **(C)** Supervised heat map of 20 transcripts differentially expressed (Kruskal–Wallis *H* test *p* < 0.05 and FDR < 0.2) between *M. tb*-tetramer^+^ T_NLM_ (light blue), T_CM_ (magenta), and T_EFF_ (purple) CD4 T cell subsets. GE is reported as delta Et values (40 − Ct values).

Twenty-two mRNA transcripts were differentially expressed between the bulk (not *M. tb*-specific) T_N_, T_SCM_, T_CM_, and T_EFF_ CD4^+^ T cell subsets at a *p*-value of <0.05 and FDR of ≤0.05 (data not shown). These bulk memory subsets could readily be distinguished from each other by PCA (Figure 1A) of these 22 transcripts. When the component loadings from principal components 1 and 2 were applied to the *M. tb*-specific tetramer^+^ cell subset data, CFP10-tetramer^+^ T_NLM_ cells clustered with bulk CD4^+^ T_SCM_ cells and were distinct from bulk CD4^+^ T_N_ cells (Figure 1A). Unsupervised hierarchical clustering based on expres-sion of these 22 transcripts also showed that CFP10-tetramer^+^ T_NLM_ cells clustered with bulk CD4^+^ T_SCM_ cells, and expressed transcripts associated with antigen-experienced cells, such as TNF-α, IFN-γ, perforin, granulysin, granzyme A and K, CCL5 (RANTES), CCR4, and CCR5. By contrast, bulk CD4^+^ T_N_ cells did not express these transcripts and formed a discrete cluster (Figure 1B).

Next, we determined whether CFP10-tetramer^+^ T_NLM_ exhibited a transcriptional profile distinct from *M. tb*-specific tetramer^+^ CD4^+^ T_CM_ and T_EFF_ memory subsets. We compared the mRNA expression between the three *M. tb*-specific tetramer^+^ CD4^+^ T cell subsets and selected 20 mRNA transcripts (*p* < 0.05 and FDR ≤ 0.2) for cluster analysis (Figure 1C). Unsupervised hierarchical clustering (Figure 1C) and PCA analysis (Figure S2 in Supplementary Material) revealed substantial overlap in GE between the CFP10-tetramer^+^ cell subsets, although CFP10-tetramer^+^ T_NLM_ appeared to cluster more closely with T_CM_ cells than T_EFF_ cells.

Since the GE profiles of *M. tb*-specific CD4^+^ cells with a naïve phenotype (CD45RA^+^ CCR7^+^ CD27^+^) were distinct from T_N_ cells and consistent with antigen-experienced, bulk T_SCM_, we concluded that these cells are *M. tb*-specific CD4^+^ T_SCM_ cells.

### *M. tb*-Specific T_SCM_ Cells Are Induced by Primary *M. tb* Infection

**Figure 2:**
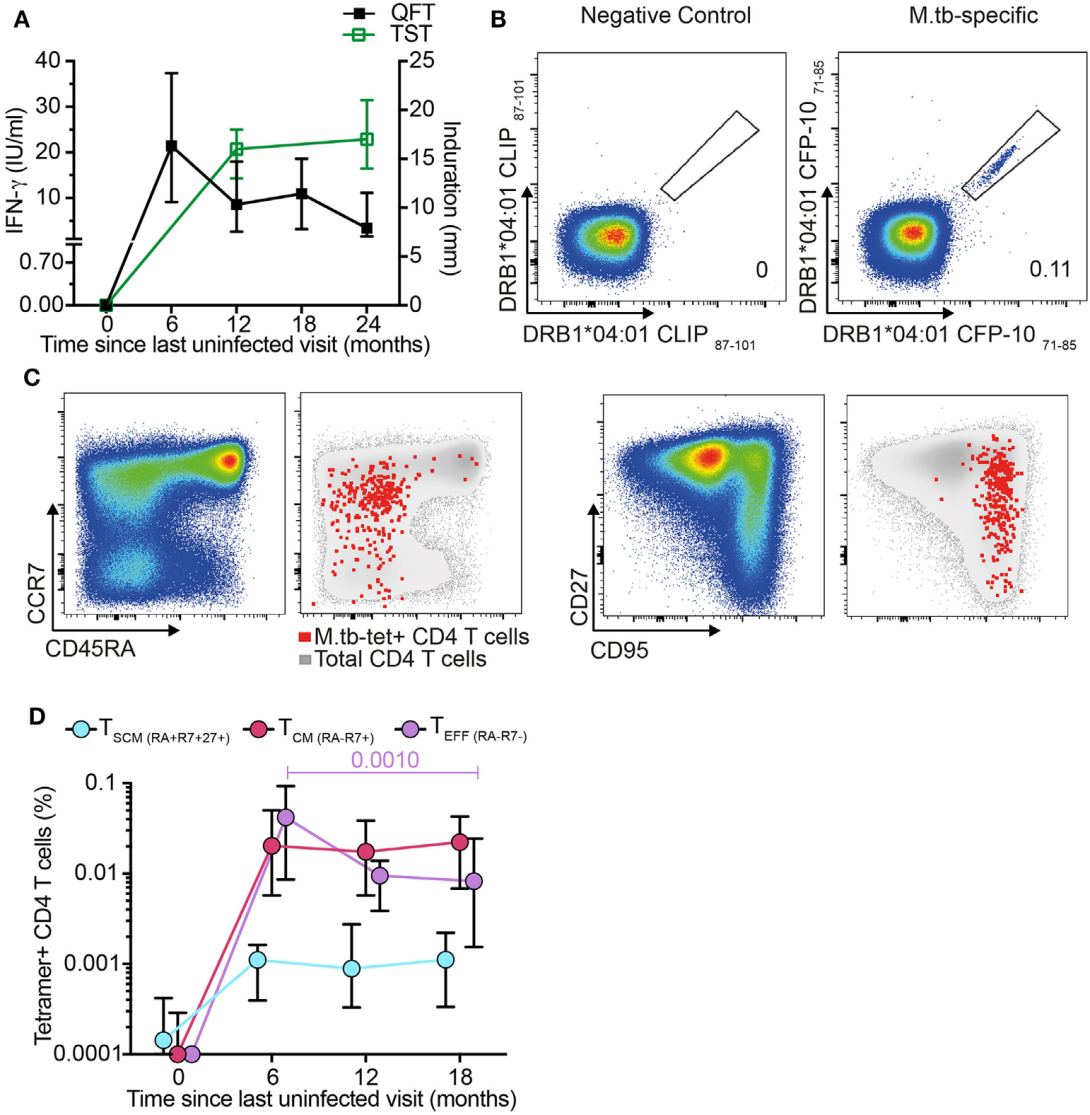
*Mycobacterium tuberculosis* (*M. tb*)-specific T_SCM_ are detected in recently *M. tb*-infected adolescents. **(A)** Median (error bars denote IQR) of Tuberculin Skin Test (TST) and QuantiFERON-TB Gold In-Tube (QFT) performed at the indicated time points. A 5 mm and 0.35 IU/ml cutoff were used to define positive TST and QFT, respectively (*n* = 19 participants). **(B)** Representative flow cytometry plots showing peripheral blood mononuclear cells staining with two identical major histocompatibility complex (MHC) class II tetramers conjugated to PE and APC to detect CD4^+^ T cells specific for CFP-10. Tetramers matched by MHC allele but loaded with a self-peptide were used as negative control. Number indicates percentage of tetramer^+^ CD4 T cells. **(C)** Representative flow cytometry plots for memory markers CD45RA, CCR7, CD27, and CD95 gated on bulk (pseudo-color and gray) and *M. tb*-specific CD4^+^ T cells (red dots). **(D)** Median (error bars denote IQR) frequencies of *M. tb*-specific T_SCM_ (CD45RA^+^ CCR7^+^ CD27^+^, light blue), T_CM_ (CD45RA^−^ CCR7^+^, magenta), and T_EFF_ (CD45RA^−^ CCR7^−^, purple) during primary *M. tb* infection (*n* = 12 participants).

We next determined whether antigen-specific CD4^+^ T_SCM_ cells are induced during primary *M. tb* infection. We retrieved stored PBMC collected from adolescents before and after *M. tb* infection, as determined by negative QFT and TST tests, with subsequent test conversion at 6- and 12-month intervals, respectively (Figure 2A). To track *M. tb*-specific CD4 T cells during acquisition of *M. tb* infection, we utilized MHC class II tetramers loaded with peptides of the *M. tb* complex-specific antigens, CFP-10 and ESAT-6. Dual staining with PE and APC-conjugated tetramers improved staining specificity (Figure 2B; Figure S3A in Supplementary Material). Frequencies of *M. tb*-tetramer^+^ CD4^+^ T cells were below the limit of reliable detection before *M. tb* infection in 11 out of 12 participants and increased significantly upon QFT conversion 6 months later, when this response also peaked. Thereafter, frequencies of *M. tb*-tetramer^+^ CD4^+^ T cells decreased but were maintained at detectable levels throughout established *M. tb* infection, at months 12 and 18 (Figure S3B in Supplementary Material).

Frequencies of *M. tb*-specific tetramer^+^ T_SCM_ (CD45RA^+^ CCR7^+^ CD27^+^), T_CM_ (CD45RA^−^ CCR7^+^), and T_EFF_ (CD45RA^−^ CCR7^−^) were below the limit for reliable detection at enrollment (month 0), demonstrating that circulating *M. tb*-specific T_N_ are too rare to detect by direct *ex vivo* tetramer staining (Figures 2C–D). After *M. tb* infection all three *M. tb*-specific memory subsets increased to detectable levels and remained detectable throughout established infection (Figure 2D). Interestingly, frequencies of *M. tb*-specific T_SCM_ and T_CM_ remained relatively consistent throughout primary and established *M. tb* infection, while *M. tb*-specific T_EFF_ peaked during the primary *M. tb* infection phase (month 6) and thereafter steadily decreased (Figure 2D). These data suggest that *M. tb*-specific T_SCM_ are induced by *M. tb* infection and maintained at low but consistent levels in the peripheral blood once *M. tb* infection has been established.

### *M. tb*-Specific T_SCM_ Express Chemokine Receptor and Cytotoxic Profiles Distinct from Bulk T_N_ and T_SCM_ CD4 T Cells

**Figure 3:**
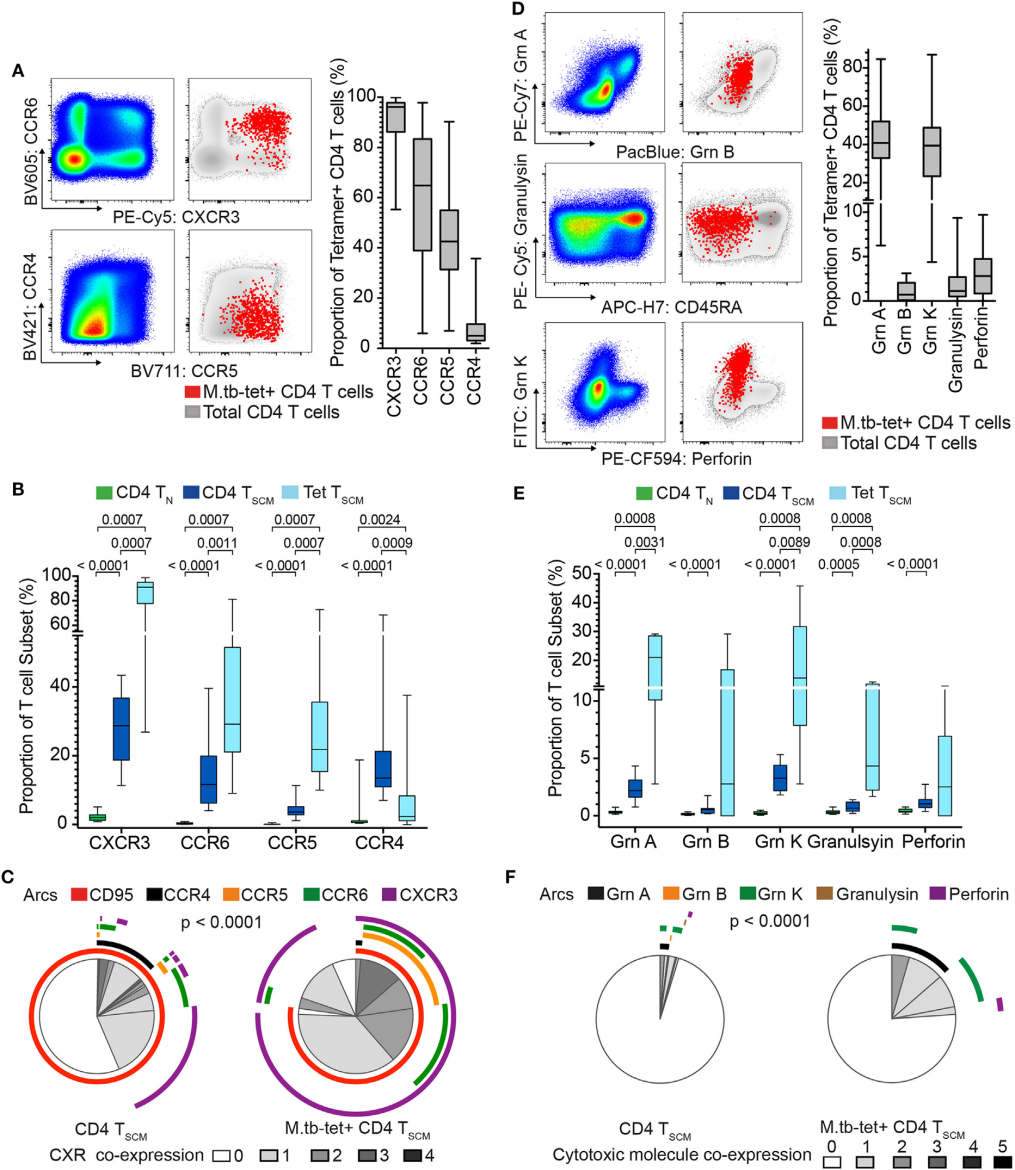
*Mycobacterium tuberculosis* (*M. tb*)-specific T_SCM_ are not T_N_ and express distinct homing and cytotoxic profiles from bulk T_SCM_. Expression of chemokine receptors and cytotoxic molecules was measured in remotely *M. tb*-infected (QFT^+^) adults (*n* = 28) when at least 20 events were detected in each tetramer^+^ memory subset. **(A)** Representative flow cytometry plots of chemokine receptor expression on bulk (pseudo-color and gray) and *M. tb*-specific (red dots) CD4^+^ T cells. Box and whisker plots represent the median proportion, IQR, and range of expression of chemokine receptor (*n* = 28). **(B)** Box and whisker plot depicting the proportion of chemokine receptor-expressing bulk T_N_ (CD45RA^+^ CCR7^+^ CD27^+^ CD95^−^, *n* = 28, green), T_SCM_ (CD45RA^+^ CCR7^+^ CD27^+^ CD95^+^, *n* = 28, blue), and *M. tb*-specific T_SCM_ (CD45RA^+^ CCR7^+^ CD27^+^, *n* = 15, light blue) CD4^+^ T cells. *p*-Values were calculated using Wilcoxon signed-rank test and corrected for multiple comparison using the Benjamini–Hochberg method with an false discovery rate (FDR) of 0.05. Adjusted *p*-values <0.05 were considered significant. **(C)** Pie chart showing the median proportions (slices) of bulk T_SCM_ (*n* = 28) and *M. tb*-specific T_SCM_ (*n* = 15) CD4^+^ T cells co-expression of CD95, CCR4, CCR5, CCR6, and/or CXCR3, denoted by arcs. *p*-Value was calculated using non-parametric permutation test comparing the overall distribution between pies. **(D)** Representative flow cytometry plots of cytotoxic molecule expression on total (pseudo-color and gray) and *M. tb*-specific (red dots) CD4^+^ T cells. Box and whisker plots represent the median proportion, IQR, and range of expression of cytotoxic molecules (*n* = 20). **(E)** Proportion of granzyme (grn) A, grnB, grnK, granulysin, and perforin expression in bulk T_N_ (*n* = 20, green), T_SCM_ (*n* = 20, blue), and *M. tb*-specific T_SCM_ (*n* = 5, light blue) CD4^+^ T cells. *p*-Values were calculated with the Mann–Whitney test, corrected for multiple comparisons with the Benjamini–Hochberg method with an FDR of 0.05. Adjusted *p*-values <0.05 were considered significant. **(F)** Pie chart showing the median proportions (slices) of bulk T_SCM_ (*n* = 20) and *M. tb*-specific T_SCM_ (*n* = 5) CD4^+^ T cells co-expression grnA, grnB, grnK, granulysin, and/or perforin, denoted by arcs. *p*-Value was calculated using non-parametric permutation test comparing the overall distribution between pies.

Our transcriptomic analysis showed that bulk CD4^+^ T_SCM_ and *M. tb*-specific T_SCM_ cells expressed chemokine receptor and cytotoxic molecule transcripts, which were mostly undetectable in bulk T_N_ cells (Figure 1B). Chemokine receptors mediate tissue homing and allow classification of memory CD4 cells into Th1, Th2, and Th17 lineages (35–37). Expression of cytotoxic mediators by CD4^+^ T cells indicates highly differentiated cells typically associated with high antigen exposure and effector T cell properties (38). We sought to validate our transcriptomic findings by measuring protein expression in an independent cohort of *M. tb*-infected (QFT^+^) adults. Expression of CCR4, CCR5, CCR6, and CXCR3 and cytotoxic molecules granzyme A, B, and K, granulysin, and perforin by *M. tb*-specific T_SCM_ (CD45RA^+^ CCR7^+^ CD27^+^) cells was compared with bulk T_N_ (CD45RA^+^ CCR7^+^ CD27^+^ CD95^−^) and T_SCM_ (CD45RA^+^ CCR7^+^ CD27^+^ CD95^+^) cells (Figure 3; Figures S1C,D in Supplementary Material).

Virtually, all *M. tb*-tetramer^+^ CD4^+^ cells expressed CXCR3 (median and IQR: 96.2 and 86.2–97.8%), with relatively high proportions also expressing CCR5 and CCR6 (Figure 3A). A very small proportion of tetramer^+^ CD4 T cells expressed CCR4 (Figure 3A). By contrast, expression of these chemokine receptors was negligible or not detected on bulk T_N_ as expected of naïve cells (Figure 3B), while CXCR3, CCR6, and CCR5 were expressed by a small proportion of bulk T_SCM_ (Figure 3B). Co-expression of these chemokine receptors by bulk T_SCM_ and *M. tb*-specific T_SCM_ revealed interesting patterns. Bulk T_SCM_ cells mostly expressed only a single chemokine receptor, whereas *M. tb*-specific T_SCM_ displayed more diverse co-expression profiles with single (CXCR3^+^), double (CCR5^+^ CXCR3^+^ or CCR6^+^ CXCR3^+^) and a small proportion of triple (CCR5^+^ CCR6^+^ CXCR3^+^) positive cells (Figure 3C). Importantly, a small subset of *M. tb*-specific T_SCM_ and >50% of bulk T_SCM_ did not express any of the chemokine receptors (Figure 3C), a profile suggesting early T cell differentiation (36). Virtually, all *M. tb*-specific CD4 T_SCM_ cells expressed CXCR3, while CD95, a typical marker of CD8 T_SCM_ cells (4, 39), was expressed by approximately 75% of tetramer^+^ CD4^+^ T_SCM_ cells (Figure 3C)—refer to Supplementary Material for factors that affected CD95 expression in our experiments (Figure S4 in Supplementary Material).

Approximately 40% (range: 4.38–86.9%) of *M. tb*-tetramer^+^ CD4 T cells expressed granzyme A and/or K. Very few *M. tb*-tetramer^+^ CD4 T cells expressed granzyme B, granulysin, or perforin (Figure 3D). Interestingly, about a quarter of *M. tb*-specific CD4^+^ T_SCM_ expressed cytotoxic molecules, also dominated by granzyme A and K. These were generally not co-expressed by *M. tb*-specific CD4^+^ T_SCM_. By contrast, bulk T_SCM_ expressed very low levels of cytotoxic molecules (Figures 3E,F).

Increased expression of some chemokine receptors and cytotoxic molecules by *M. tb*-specific T_SCM_ compared with bulk T_SCM_ suggested that *M. tb*-specific T_SCM_ are more phenotypically differentiated than bulk T_SCM_. We thus also compared chemokine receptor and cytotoxic molecule expression patterns by *M. tb*-specific T_SCM_ with bulk T_CM_ (CD45RA^−^ CCR7^+^ CD27^+^) and T_EFF_ (CD45RA^−^ CCR7^−^CD27^−^) cells (Figures S5A,B in Supplementary Material). *M. tb*-specific T_SCM_ expressed higher levels of CXCR3 than both bulk T_CM_ and T_EFF_ cells, further supporting their T_SCM_ identity. Surprisingly, *M. tb*-specific T_SCM_ had similar expression levels of CCR5 and CCR6 to bulk T_EFF_ cells, but significantly lower expression of CCR4 than bulk T_CM_ or T_EFF_ cells (Figure S5A in Supplementary Material). *M. tb*-specific T_SCM_ also expressed significantly higher levels of granzyme A and K than bulk T_CM_ (Figure S5B in Supplementary Material). However, T_SCM_ expressed lower levels of all cytotoxic molecules except granzyme K than bulk T_EFF_ cells. These data further support the finding that *M. tb*-specific T_SCM_ cells are antigen-experienced memory cells with unique phenotypic and functional attributes that distinguish them from bulk T_SCM_ cells, and are more similar to highly differentiated bulk T_CM_ and T_EFF_ cells.

### *M. tb*-Specific CD4 T_SCM_ Are Less Differentiated than *M. tb*-Specific T_CM_ and T_EFF_ CD4 T Cells

**Figure 4:**
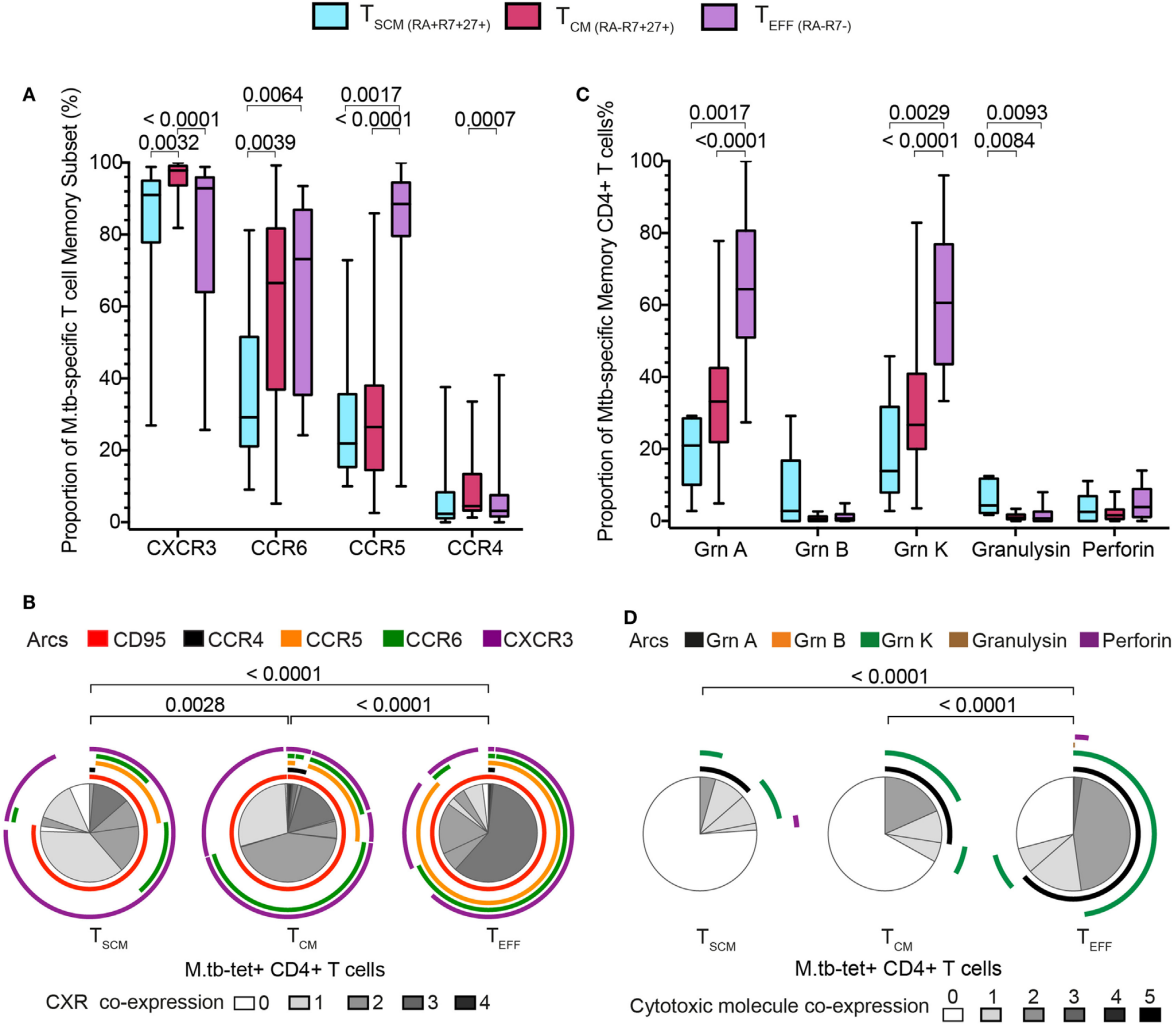
*Mycobacterium tuberculosis* (*M. tb*)-specific T_SCM_ display early memory tissue homing and cytotoxic profiles. Expression of chemokine receptors and cytotoxic molecules was measured in remotely *M. tb*-infected (QFT^+^) adults (*n* = 28) when at least 20 events were detected in each tetramer^+^ memory subset. **(A)** Box and whiskers plots depicting the proportion of chemokine receptors expression of *M. tb*-specific T_SCM_ (*n* = 15, light blue), T_CM_ (*n* = 27, magenta), and T_EFF_ (*n* = 23, purple) CD4^+^ T cells. *p*-Values were calculated using Wilcoxon signed-rank test and corrected for multiple comparison using the Benjamini–Hochberg method with a false discovery rate (FDR) of 0.05. Adjusted *p*-values <0.05 were considered significant. **(B)** Pie chart showing the median proportions (slices) of *M. tb*-specific T_SCM_ (*n* = 15), T_CM_ (*n* = 27), and T_EFF_ (*n* = 23) CD4^+^ T cells co-expression of CD95, CCR4, CCR5, CCR6, and/or CXCR3, denoted by arcs. *p*-Values were calculated using non-parametric permutation test comparing the overall distribution between pies. **(C)** Box and whiskers plots depicting the proportion of grnA, grnB, grnK, granulysin, and perforin expression in *M. tb*-specific T_SCM_ (*n* = 5, light blue), T_CM_ (*n* = 19, magenta), and T_EFF_ (*n* = 17, purple) CD4^+^ T cells. *p*-Values were calculated using Mann–Whitney test and corrected for multiple comparison using the Benjamini–Hochberg method with an FDR of 0.05. Adjusted *p*-values <0.05 were considered significant. **(D)** Pie chart showing the median proportions (slices) of *M. tb*-specific T_SCM_ (*n* = 5), T_CM_ (*n* = 19), and T_EFF_ (*n* = 17) CD4^+^ T cells co-expression of grnA, grnB, grnK, granulysin, and/or perforin, denoted by arcs. *p*-Values were calculated using non-parametric permutation test comparing the overall distribution between pies.

We then compared the expression of chemokine receptors and cytotoxic molecules between *M. tb*-specific tetramer^+^ CD4^+^ T_SCM_ cells and the other tetramer^+^ CD4^+^ T cell memory subsets. Virtually all cells among the three *M. tb*-specific memory subsets expressed CXCR3, as has been previously reported for *M. tb*-specific CD4^+^ T cells, although T_CM_ displayed the highest proportion of CXCR3^+^ cells, compared with *M. tb*-specific T_SCM_ and T_EFF_ cells (Figure 4A). A significantly lower proportion of *M. tb*-specific T_SCM_ expressed CCR6 than either *M. tb*-specific T_CM_ or T_EFF_ cells, while a minority of *M. tb*-specific T_SCM_ and T_CM_ expressed CCR5 or CCR4. As expected, virtually all *M. tb*-specific T_EFF_ cells were CCR5^+^ and CCR4^−^. An increase from single to triple chemokine receptor co-expression profiles was observed when *M. tb* memory cells were ordered according to their expected differentiation sequence, from T_SCM_ to T_CM_ to T_EFF_ cells (Figure 4B; Figure S5A in Supplementary Material). This was also observed when expression of the cytotoxic molecules granzyme A and K was assessed. Proportions of *M. tb*-specific T_SCM_ cells expressing granzyme A and/or K were lower than T_CM_ expressing these cytotoxic molecules, which in turn were lower than *M. tb*-specific T_EFF_ cells (Figure 4C). Proportions of *M. tb*-specific T_EFF_ expressing both granzyme A and K were also higher than those observed in *M. tb*-specific T_CM_ and T_SCM_ CD4^+^ T cells (Figure 4D; Figure S5B in Supplementary Material).

Taken together, these data show that *M. tb*-specific T_SCM_ pos-sess the least differentiated *M. tb*-specific phenotypic and functional profile, and suggest that *M. tb*-specific T_CM_ cells appear as an intermediate subset before cells differentiate into *M. tb*-specific T_EFF_ CD4^+^ T cells.

### *M. tb*-Specific T_SCM_ CD4 T Cells Express Th1 Cytokines

**Figure 5:**
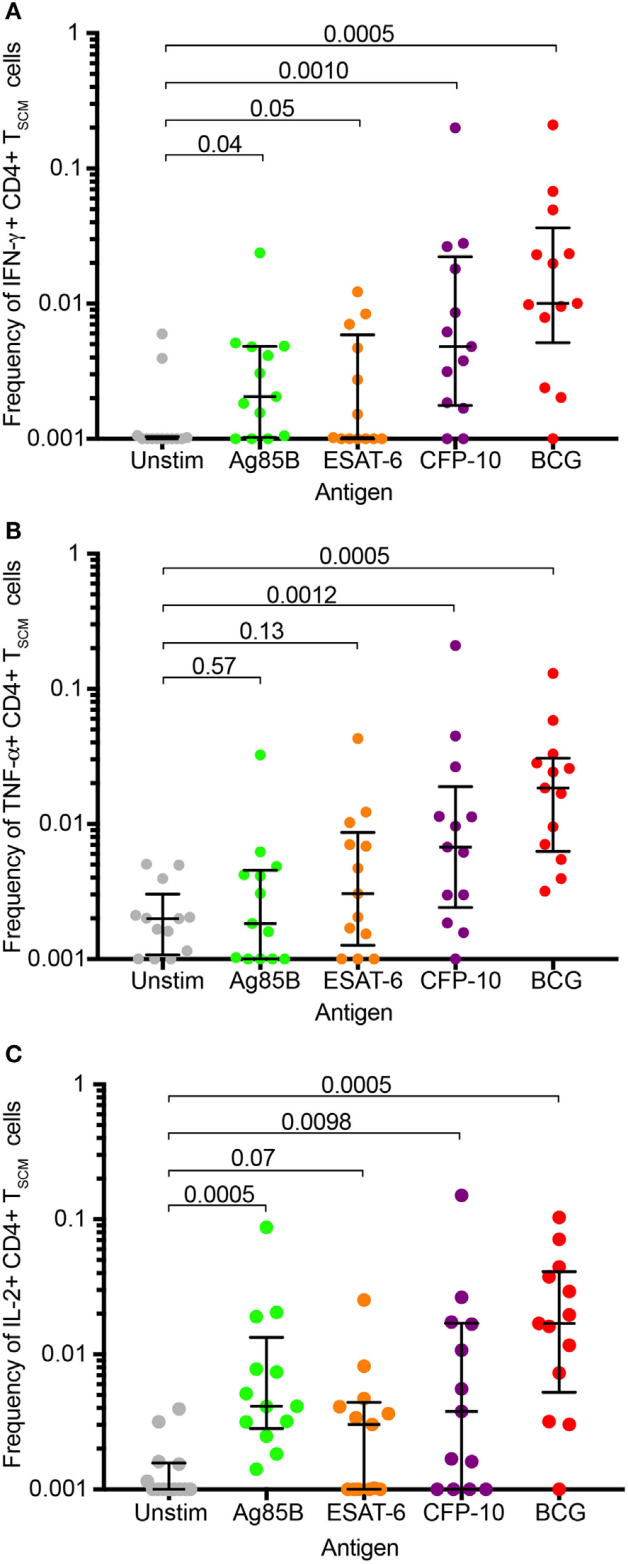
*Mycobacterium tuberculosis* (*M. tb*)-specific T_SCM_ express Th1 cytokines. Fresh whole blood from remotely *M. tb*-infected (QFT^+^) adults (*n* = 13) was left unstimulated (gray) or stimulated with peptide pools spanning *M. tb* antigens, Ag85B (green), ESAT-6 (orange), or CFP-10 (purple), or whole bacillus Calmette–Guerin (red) for 12 h. Box and whisker plots depict frequencies of CD4^+^ T_SCM_ (RA^+^ R7^+^) T cells expressing IFN-γ **(A)**, TNF-α **(B)**, or IL-2^+^ **(C)**. *p*-Values were calculated using the Wilcoxon matched pairs test, and *p*-values <0.0125 were considered significant (corrected for multiple testing using the Bonferroni method).

Studies of CD8^+^ (and CD4^+^) T_SCM_ have shown that T_SCM_ have limited cytokine expression capacity that is dominated by IL-2 and TNF-α in responses to non-specific stimulation (2, 3, 5). To determine if this is also true for mycobacteria-specific T_SCM_, we re-analyzed available data from stimulated whole blood (WB-ICS, Figure S6A in Supplementary Material) from a previously published cohort of QFT^+^ adults (28). Significantly higher frequencies of IFN-γ, TNF-α, or IL-2 cytokine-expressing CD4 T_SCM_ cells, defined as CD45RA^+^ CCR7^+^, were detected in blood stimulated with BCG, or peptide pools spanning either Ag85B or CFP-10, compared with unstimulated blood (Figure 5)—refer to Supplementary Information for the justification for using CD45RA^+^ CCR7^+^ as a marker for T_SCM_ by cytokine-expressing CD4^+^ T cells (Figure S7 in Supplementary Material). This was, however, not observed in blood stimulated with ESAT-6.

In summary, cytokine production by mycobacteria-specific CD4^+^ T_SCM_ cells is not restricted to IL-2 but includes TNF-α and IFN-γ.

### BCG-Specific T_SCM_ Cell Frequencies Are Associated with CD4^+^ T Cell Proliferative Capacity

**Figure 6:**
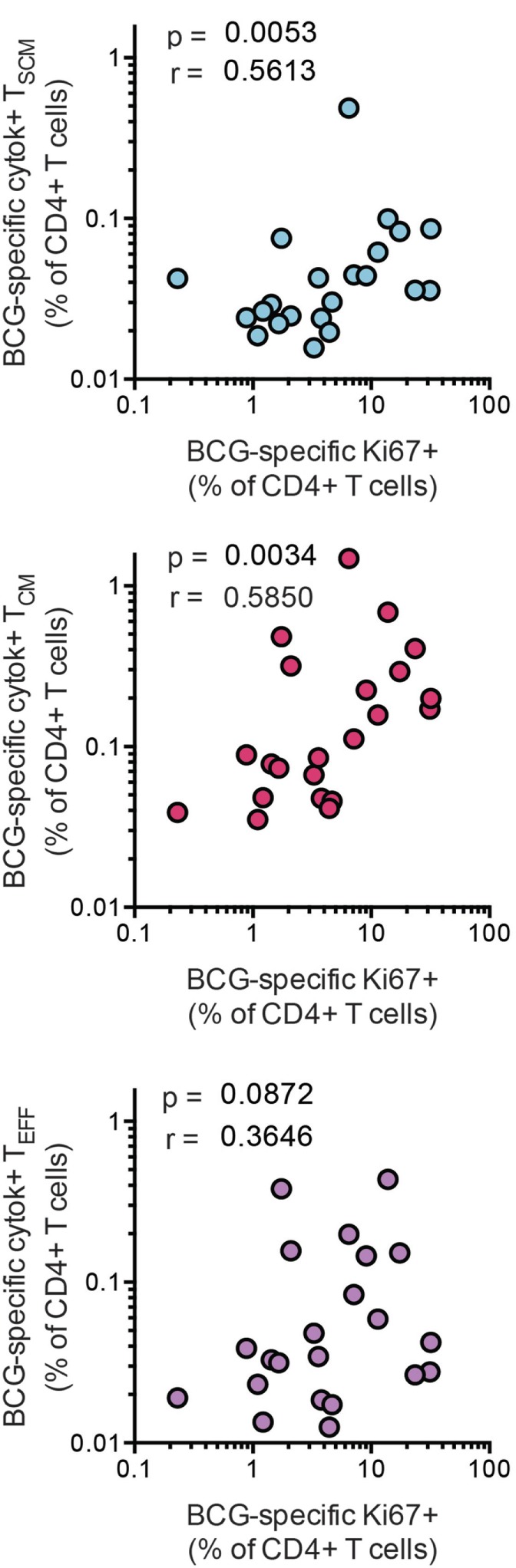
Bacillus Calmette–Guerin (BCG)-specific T_SCM_ are associated with long-term CD4^+^ T cell proliferation after vaccination. Whole blood from 1-year-old infants (*n* = 23) was stimulated with BCG for 12 h to measure the frequencies of cytokine-producing T_SCM_ (CD45RA^+^, CCR7^+^), T_CM_ (CD45RA^−^, CCR7^+^), and T_EFF_ (CD45RA^−^, CCR7^−^). In parallel, whole blood was stimulated with BCG for 7 days, and the frequency of proliferating CD4^+^ cells was assessed by upregulation of Ki-67. Correlations between the frequencies of BCG-specific CD4^+^ memory T cell subsets and those of proliferating CD4^+^ T cells were calculated by Spearman test 10 months postvaccination.

A key feature of T_SCM_ is long-term maintenance of proliferative capacity in the absence of antigenic stimulation (2). To determine whether vaccine-induced CD4^+^ T_SCM_ contribute to T cell long-term proliferative potential, we analyzed available data from a recently completed clinical trial in infants, who received BCG vaccination (26). Frequencies of BCG-specific CD4^+^ T_SCM_ and T_CM_ cells, but not T_EFF_ cells, positively correlated with proliferative potential of BCG-stimulated CD4^+^ T cells 10 months after BCG vaccination (Figure 6). These results suggest that vaccine-induced T_SCM_ contribute to long-term memory and proliferative capacity of the vaccine-induced T cell response to mycobacteria.

## Discussion

We performed in-depth analyses of the kinetics, phenotype and functional characteristics of *M. tb*-specific CD4^+^ T_SCM_ cells during natural *M. tb* infection. Our data show that CD4^+^ T_SCM_ induced by *M. tb* infection in humans, predominantly express CD95 and CXCR3, are distinct from naïve T cells and possess phenotypic and functional profiles consistent with *M. tb*-specific CD4^+^ T cells at an early stage of differentiation (36). These data supplement our current knowledge of T_SCM_ cells, which has primarily been derived from studies of virus-specific CD8 T cells (2–4, 7).

CD4^+^ *M. tb*-specific T_SCM_ were induced during primary *M. tb* infection and maintained throughout established *M. tb* infection at low frequencies. This phenomenon is highly characteristic of the stem cell nature of long-term memory cells such as T_CM_ and potentially T_SCM_ cells, where asymmetrical cell division maintains both the overall proportions of these memory cells and the pool of more differentiated effector memory subsets (40, 41).

The transcriptomic profile of *M. tb*-specific T_SCM_ cells overlapped substantially with bulk CD4^+^ T_SCM_. However, protein expression of chemokine receptors and cytotoxic molecules distinguished bulk from *M. tb*-specific T_SCM_. Our data show that *M. tb*-specific T_SCM_ cells possess unique phenotypic and functional profiles that share more similarities with bulk T_CM_ and T_EFF_ memory cells than bulk T_SCM_ cells. This might suggest that *M. tb*-specific T_SCM_ are exposed to chronic antigen stimulation, which is probably not the case for all T_SCM_ specific for other pathogens, resulting in a more differentiated chemokine and cytotoxic molecules expression pattern. Whether this increased phenotypic differentiation profile is unique to *M. tb*-specific CD4 T_SCM_ cells requires further investigation. Consistent with published findings (21), we found that *M. tb*-specific cells were predominantly CXCR3^+^, a Th1 associated chemokine receptor (42). This was accompanied with relatively high CCR5 and CCR6 expression, chemokine receptors associated with activation (43) and Th1/17 T cells (44), respectively, but low expression of the Th2 associated CCR4 (42). The predominant CXCR3 expression by *M. tb*-specific T_SCM_ is suggestive of Th1 lineage, which we confirmed on a functional level by showing that mycobacteria-specific T_SCM_ not only produced, IL-2 but also TNF-α and IFN-γ and, to a lower extent, cytotoxic molecules.

Our data suggested that *M. tb*-specific T_SCM_ had different cytokine expression patterns depending on the *M. tb* antigen recognized. For example, Ag85B-specific T_SCM_ produced only IL-2, while BCG- and CFP-10-specific T_SCM_ produced TNF-α and IFN-γ in addition to IL-2. Despite the early stage of T cell memory differentiation of T_SCM_ these findings may reflect different degrees of antigen exposure *in vivo*. A murine study showed that Ag85B mRNA expression peaks during early stages of *M. tb* infection and significantly reduces during established infection, whereas ESAT-6 mRNA was maintained at high levels throughout these infection stages (45). We also recently showed differential degrees of T cell differentiation of Ag85B and ESAT-6-specific CD4 T cells in *M. tb*-infected mice and humans (46). Expression of only IL-2, a cytokine associated with homeostatic proliferation and early T cell differentiation, by Ag85B-specific T_SCM_ thus may reflect lower exposure of T cells to this antigen. By contrast, expression of TNF-α and IFN-γ, cytokines typically produced by more differentiated T cells, by CFP-10 and BCG-specific T_SCM_ is consistent with higher *in vivo* recognition or exposure to these antigens (36). However, in light of the low frequencies of *M. tb*-specific T_SCM_ these data on functional profiles should be interpreted conservatively. The nature of cytokine expression by *M. tb*-specific T_SCM_ cells requires more attention in future studies. In addition, co-expression of CCR6, observed in about a third of *M. tb*-specific T_SCM_, might indicate early polarization toward Th1/Th17 lineage, which has been described as the major CD4^+^ T cell subset responding to *M. tb* (21). Finally, CCR5 expression, observed in about 20% of *M. tb*-specific T_SCM_, suggests that at least some *M. tb*-specific T_SCM_ may have the potential to migrate to sites of infection (47, 48) and are not confined to the lymphoid compartment, despite CCR7 expression.

The ability of CD4^+^ T cells to produce cytotoxic molecules has been associated with high antigen exposure resulting in increased MHC-antigen-T cell receptor interaction and is mostly associated with late differentiated T cells with increased effector functions (38, 49, 50). Cytotoxic CD4^+^ T cells have been well characterized in contexts of viral infections, where cytotoxic CD4^+^ T cells either utilize granzyme B and perforin or Fas–FasL interactions to mediate killing of infected cells (49, 50). Surprisingly, we found that CD4^+^ *M. tb*-specific T cells, including *M. tb*-specific T_SCM_ cells, predominantly expressed granzyme A and/or K. Unlike granzyme B and perforin that mediate cytotoxic killing of infected cells, granzymes A and K, have been described as pro-inflammatory and cytokine-inducing cytotoxic molecules (51–54). In fact, expression of granzyme A by γδ T cells mediated increased *M. tb* killing by macrophages (55). Thus, expression of these cytotoxic molecules by *M. tb*-specific T_SCM_, T_CM_, and T_EFF_, in absence of granzyme B and perforin, may induce pro-inflammatory responses in macrophages at the site of infection, rather than mediate direct killing of *M. tb*-infected macrophages, which would require perforin to mediate entry of granzymes into infected cells.

We primarily employed MHC class II tetramers loaded with *M. tb*-epitopes to identify *M. tb*-specific CD4^+^ T cells directly *ex vivo* for transcriptional and phenotypic profiling (Figures S1A–D in Supplementary Material). MHC tetramers provide a method for detecting antigen-specific T cells directly *ex vivo* without the need for antigen stimulation, which can alter the function, phenotype and GE profiles of T cells. However, an important limitation to using MHC class II tetramers is that only T cells that bear the cognate TCR for a single peptide in the context of a single MHC allele can be studied. To address this, we employed several tetra-mers that allowed detection of CFP-10, ESAT-6, and Ag85-specific CD4^+^ T cells restricted by DRB1*0301, DRB1*0401, DRB5*0101, DPB1*0401, or DQB*0602, providing adequate coverage of the *M. tb*-specific CD4 T cell response and cohort. Another limita-tion of our study was our inability to characterize the proliferation and differentiation potential of purified *M. tb*-specific CD4^+^ T_SCM_ cells, because very large number of cells (approximately 500 million PBMC per participant) would be required to sort at least 1,000 *M. tb*-specific tetramer^+^ CD4 T_SCM_ cells. This is only achievable by leukapheresis, which was not available at our clinical site, where study visits were performed. We provided indirect evidence for this by showing that the precursor frequencies of BCG-specific T_SCM_ and T_CM_ CD4^+^ T cells correlated with proliferative potential of BCG-specific CD4^+^ T cells 10 months post infant BCG vaccination. Further studies measuring vaccine-induced T_SCM_ and long-term persistence of memory T cells are required to define the role of antigen-specific CD4^+^ T_SCM_ after vaccination.

In summary, we have shown that *M. tb*-specific CD4^+^ T_SCM_ are induced by primary *M. tb* infection and exhibit transcriptional, phenotypic and functional features that are distinct from bulk CD4^+^ T_N_ and T_SCM_ and consistent with early differentiation of *M. tb*-specific CD4^+^ T cells, possibly shaped by antigen exposure. Our findings have important implications in the study of the *M. tb*-specific T cell memory repertoire and raise numerous unanswered questions. For example, determining the functional role of *M. tb*-specific T_SCM_ in long-term immune responses, how their features differ between asymptomatic *M. tb* infection and TB disease, as well as during TB treatment. In addition, future work should determine whether long-lived CD4^+^ T_SCM_ can be induced by vaccination, and whether they are associated with long-term memory responses and protection, as observed for CD8^+^ T_SCM_ upon yellow fever vaccination (7) as well as in adoptive immune-therapy (4, 8, 9).

## The SATVI Clinical Immunology Team

**Cynthia Ontong**, **Elizabeth Filander**, **Fadia Alexander**, **Hadn Africa**, **Janelle Botes**, **Lebohang Makhethe**, **Lungisa Jaxa**, **Marcia Steyn**, **Noncedo Xoyana**, **Rachel Oelfose**, **Sindile Matiwane**, South African Tuberculosis Vaccine Initiative, Institute of Infectious Disease and Molecular Medicine, Division of Immunology, Department of Pathology, University of Cape Town, Cape Town, South Africa.

## Ethics Statement

Consent forms and study protocols were approved by the Human Research Ethics Committee of the University of Cape Town (UCT HREC 126/2006, 045/2008, 179/2011, 013/2012, 753/2014). All adult participants provided written informed consent. Parents or legal guardians of adolescents provided written informed consent and adolescents provided written informed assent.

## Author Contributions

CAMM, OBD, MH, EN, and TJS contributed to conception and design of the study. CAMM and OBD performed experimental work. SM, NB, EN, and TJS contributed to execution and oversight of experimental work. CM and MM performed statistical analyses. CAMM, OBD, MM, EN, and TJS contributed to data interpretation and drafted the manuscript. All the authors read, revised, and approved the final version of the manuscript.

## Conflict of Interest Statement

The authors declare that the research was conducted in the absence of any commercial or financial relationships that could be construed as a potential conflict of interest.
