# Supplementary material for: Functional, Antigen-Specific Stem Cell Memory (TSCM) CD4+ T Cells Are Induced by Human Mycobacterium tuberculosis Infection
Source: Front Immunol. 2018 Mar 1;9:324. doi: 10.3389/fimmu.2018.00324 (PMC5839236; doi:10.3389/fimmu.2018.00324)
Supplement: Supplementary file 1 [file Table_1.PDF]

Supplementary Table 1: Antibodies used to detect memory markers, chemokine receptors, cytotoxic molecules and cytokines expression profiles of CD4+ T cells.

Marker	Clone	Manufacturer	Flourochrome	Sample Type	Staining	Panel
CCR4	1G1	BD Biosciences	BV421	Fresh PBMC	Surface	3
CCR5	2D7	BD Biosciences	BV711			
CCR6	11A9	BD Biosciences	BV605			
CCR7	150503	BD Biosciences	BV711	Cryopreserved PBMC		4
			PerCP Cy5.5	Cryopreserved PBMC		1
			PE-CF594	Fresh/Cryopreserved PBMC		2-3
	150503	eBioscience	PE	Cryopreserved whole blood	Intracellular	5
CXCR3	1C6/CXCR3	BD Biosciences	PE-Cy 5	Fresh PBMC	Surface	3
CD3	UCHT1	BD Biosciences	Alexa Fluor 700	Fresh/Cryopreserved PBMC		1-4
	MOPC-21	BD Biosciences	BV 431	Cryopreserved whole blood	Intracellular	5
CD4	L200	BioLegend	BV785	Fresh/Cryopreserved PBMC	Surface	2-3
	SK3	BD Bioscience	BV786	Cryopreserved PBMC		4
	S3.5	Invitrogen	Q-dot 605			1
				Cryopreserved whole blood	Intracellular	5
CD45RA	HI100	BD Biosciences	APC H7	Cryopreserved PBMC	Surface	4
	HI100	eBioscience	PE-Cy 7			1
	HI100	BioLegend		Fresh/Cryopreserved PBMC		2-3
	HI100	eBioscience	BV570	Cryopreserved whole blood	Intracellular	5
CD27	L128	BD Biosciences	BV650	Fresh/Cryopreserved PBMC	Surface	2-4
	M-T271		FITC	Cryopreserved PBMC		1
CD95	DX2	Biolegend	APC			
		BD Biosciences	BV605			4
			FITC	Fresh/Cryopreserved PBMC		2-3
HLA-DR	L243	BioLegend	BV421	Cryopreserved PBMC		2
CD8	SK1	BD Biosciences	PerCP-Cy5.5	Cryopreserved whole blood	Intracellular	5
CD8	RPA-T8	BD Biosciences	BV421	Cryopreserved PBMC	Surface	1
CD14	M5E2	BioLegend				
CD19	HIB19	BD Biosciences				
CD8	RPA-T8	BioLegend	BV510	Fresh/Cryopreserved PBMC		2-4
CD14	M5E2	BioLegend				
CD19	SJ25C1	BD Biosciences				
Granzyme A	CB9	eBioscience	PE-Cy 7	Cryopreserved PBMC	Intracellular	4
Granzyme B	GB11	BioLegend	Pac Blue			
Granzyme K	GM6C3	Santa Cruz Biotechnology	FITC			
Granulysin	PolyClonal (Ref AF3138)	R & D Systems	PE-Cy 5 (in-house conjugation)			
Perforin	δG9	BD Biosciences	PE-CF594			
IL-2	5344.111	BD Biosciences	FITC	Cryopreserved whole blood		5
IL-17	SCPL1362	eBioscience	Alexa Fluor 647			
IFN-	B27	BD Biosciences	Alexa Fluor 700			
TNF-	Mb11	eBioscience	PE-Cy 7			
Live/Dead	N/A	Thermo-Fisher Scientific	ViViD	Cryopreserved PBMC	Surface	1
Live/Dead	(Ref L34957)	Thermo-Fisher Scientific	Aqua	Fresh/Cryopreserved PBMC	Surface	2-4
