# Supplementary material for: Functional, Antigen-Specific Stem Cell Memory (TSCM) CD4+ T Cells Are Induced by Human Mycobacterium tuberculosis Infection
Source: Front Immunol. 2018 Mar 1;9:324. doi: 10.3389/fimmu.2018.00324 (PMC5839236; doi:10.3389/fimmu.2018.00324)
Supplement: Supplementary file 2 [file Table_2.PDF]

Supplementary Table 2: TaqMan assays used to determine T cell gene expression profiles

Gene	Applied biosystems assay	Gene	Applied biosystems assay	Gene	Applied biosystems assay	Gene	Applied biosystems assay
**Activation**		**Chemokine Receptor**		**Effector Molecules**		**Transcription Factor**	
CD154	Hs00163934_m1	CCR2	Hs01560352_m1	GNLY	Hs00246266_m1	FOXO3A	Hs00818121_m1
CD38	Hs01120071_m1	CCR4	Hs00747615_s1	GZMA	Hs00989184_m1	FOXP3	Hs01085834_m1
CD69	Hs00934033_m1	CCR5	Hs00152917_m1	GZMB	Hs01554355_m1	NFKB1	Hs00765739_m1
ICOS	Hs00359999_m1	CCR6	Hs01890706_s1	GZMK t	Hs00157878_m1	RORA	Hs00536545_m1
KI67	Hs01032443_m1	CCR7	Hs01013469_m1	PRF1	Hs00169473_m1	RORC	Hs01076112_m1
TFRC	Hs00951083_m1	CCR9	Hs01890924_s1			RHOH	Hs00180265_m1
		CXCR3	Hs01847760_s1	**Homing/Cell Trafficking**		STAT1	Hs01013996_m1
**Apoptosis**		CXCR6	Hs00174843_m1	ITGAX	Hs01015070_m1	STAT3	Hs01047580_m1
BCL2	Hs00608023_m1			SELL	Hs00174151_m1	STAT4	Hs01028017_m1
BCL2L11	Hs00708019_s1	**Cytokine Receptor**		CD44	Hs01075861_m1	STAT5A	Hs00234181_m1
BTLA	Hs00699198_m1	IFNGR1	Hs00988304_m1	CD31	Hs00169777_m1	STAT5B	Hs00273500_m1
CD137	Hs00155512_m1	IL12RB2	Hs01548202_m1	B-CATENIN	Hs00355049_m1	STAT6	Hs00598625_m1
FAS	Hs00236330_m1	IL2RA	Hs00907779_m1	ARHGEF18	Hs00248726_m1	TBX21	Hs00894392_m1
PDCD1	Hs01550088_m1	IL2RB	Hs01081697_m1			TCF3	Hs00413032_m1
		IL2RG	Hs00953624_m1	**Kinases/ receptor Kinase**		TCF7L2	Hs01009044_m1
		IL7R	Hs00902334_m1	CAMK4	Hs00174318_m1	TGFB1	Hs00998133_m1
		IL7R	Hs00902334_m1	EPHA4	Hs00177874_m1	GATA3	Hs00211122_m1
**Gattinoni/Appay**				ITK	Hs00950634_m1	LEF1	Hs01547250_m1
Glob	Hs00364202_s1	**Cytokines/Chemokine**		PRKCA	Hs00925195_m1	TCF7	Hs00175273_m1
GPR15	Hs00922903_s1	IFNG	Hs00989291_m1	JNK	Hs00177083_m1		
ENTPD1	Hs00969559_m1	IL10	Hs00961622_m1			**Housekeeper**	
FCER1G	Hs00175408_m1	IL2	Hs00174114_m1	**Co-stimulatory molecules**		B2M	Hs00984230_m1
IGF1R	Hs00609566_m1	IL4	Hs00929862_m1	CD27	Hs00386811_m1	G6PD	Hs00166169_m1
FAM129A	Hs00223000_m1	IL6	Hs00985639_m1	CD28	Hs00174796_m1	GAPDH	Hs02758991_g1
PRR5L	Hs01029928_m1	TNFA	HS01113624_g1			HPRT	Hs01003267_m1
GFPT2	Hs01049561_m1	LTB	Hs00242737_m1	**Miscellaneous**			
BAG-1	Hs00185390-m1	CXCL10	Hs01124251_g1	MAN1C1	Hs00220595_m1	**Inhibition**	
JUNB	Hs00357891_s1					CTLA4	Hs03044418_m1
IPLA2	Hs00185926_m1	**Lineage Markers**				SOCS1	Hs00705164_s1
TIMD4	Hs00293316_m1	CD4	Hs01058407_m1			CD200	Hs01033303_m1
CCL5	Hs00174575_m1	CD8A	Hs00233520_m1			AXIN2	Hs00610344_m1
