# Supplementary material for: Functional, Antigen-Specific Stem Cell Memory (TSCM) CD4+ T Cells Are Induced by Human Mycobacterium tuberculosis Infection
Source: Front Immunol. 2018 Mar 1;9:324. doi: 10.3389/fimmu.2018.00324 (PMC5839236; doi:10.3389/fimmu.2018.00324)
Supplement: Supplementary file 3 [file Data_Sheet_1.PDF]

Supplementary Material

FUNCTIONAL, ANTIGEN-SPECIFIC STEM CELL MEMORY (T_{SCM}) CD4+ T CELLS ARE INDUCED BY HUMAN *M. TUBERCULOSIS* INFECTION

Cheleka AM Mpande[1#], One B Dintwe[1#], Munyaradzi Musvosvi[1], Simbarashe Mabwe[1], Nicole Bilek[1], Mark Hatherill[1], Elisa Nemes[1*] and Thomas J Scriba[1*] and the SATVI Clinical Immunology Team[$].

[#]Theses authors contributed equally.
*These authors contributed equally.
[$]The SATVI Clinical Immunology Team: Cynthia Ontong[1], Elizabeth Filander[1], Rachel Oelfose[1], Noncedo Xoyana[1], Sindile Matiwane[1], Lungisa Jaxa[1], Janelle Botes[1], Fadia Alexander[1], Hadn Africa[1], Marcia Steyn[1], Lebohang Makhethe[1]

Correspondence: Thomas J Scriba: thomas.scriba@uct.ac.za

Supplementary Information

Detection of M.tb-specific CD4+ T cells by MHC class II tetramers

Studies of T cell functionality in humans typically utilise antigen stimulation and functional outcomes to detect antigen-specific CD4 T cell responses. Stimulation of T cells to induce expression of such functional outcomes may affect transcriptional profiles and expression of activation and inducible phenotypic markers, presenting potential confounders when studying T cells. The use of MHC tetramers provides an alternative method of detecting Ag-specific T cells directly *ex-vivo*, without the need for antigen stimulation. This technique thus provides a better representation of *in-vivo* T cell profiles than *ex-vivo* antigen stimulation. Therefore, we primarily used MHC class II tetramers (loaded with M.tb-epitopes to identify M.tb-specific CD4+ T cells and determine their transcriptional and phenotypic profiles, directly *ex-vivo* (Figure SI1A-D).

CD95 expression on M.tb-tetramer+ and M.tb-specific T_{SCM} is significantly affected by staining protocol and/or cryopreservation.

We compared PBMC that were analysed fresh (without cryopreservation) by surface staining for chemokine receptor analysis, or after cryopreservation by intra-cellular staining for analysis of cytotoxic molecule expression, using different antibody panels. This allowed analysis of the effects of cryopreservation and the staining protocols on the detection of phenotypic markers (SI4A). Our results show that cryopreservation and staining protocol significantly affected detected levels of CD45RA (higher on cryopreserved cells), CCR7 (lower on cryopreserved cells) and CD95 (markedly lower on cryopreserved cells), but not CD27 on M.tb-tetramer+ CD4+ cells, which resulted in detecting different proportions of M.tb-tetramer+ CD4+ memory subsets (SI4B). To mimize the consequences of these confounding effects on our experiments, we restricted phenotypic and functional analyses to samples containing more than 20 cells for each memory subset. Expression of CD95, a typical marker of T_{SCM} (Gattinoni *et al.*, 2011), showed the highest variability between the different methodologies (SI4C). We therefore used CD45RA+CCR7+ or CD45RA+CCR7+CD27+ CD4+ T cells, to define T_{SCM} CD4+ T cells.

M.tb-tetramer+ T_{SCM} can be defined as CD45RA+CCR7+ or CD45RA+CCR7+CD27+.

To determine if measuring CD27 expression in combination with CD45RA and CCR7 is critical for defining M.tb-specific T_{SCM} CD4+ T cells, or whether CD45RA and CCR7 in the absence of CD27 is sufficient, we compared protein expression levels (median fluorescence intensity) of the T_{SCM} markers CD27, CD95 and CXCR3 between CD45RA+CCR7+ and CD45RA+CCR7+CD27+ M.tb-tetramer+ T_{SCM} cells (Figure SI7). No differences in the expression of these T_{SCM} markers were observed between the M.tb-specific T_{SCM} cells defined by the two phenotypes, suggesting that CD45RA and CCR7 are sufficient to define CD4+ M.tb-specific T_{SCM}.

Supplementary Figure Legends

Supplementary Figure 1: Gating strategy for M.tb-tetramer+ sorting and functional characterisation.
Cryopreserved PBMC from M.tb-infected adults **(A)** and recently M.tb-infected adolescents **(B)** were gated according to the illustration. M.tb-tetramer+ responses were defined on CD4+CD3+CD8-CD14-CD19- live lymphocytes. Memory markers gates were defined on bulk CD4+ T cells and applied to tetramer+ cells, as shown. Freshly isolated **(C)** and cryopreserved **(D)** PBMC from remotely M.tb-infected adults were gated according to the illustration. M.tb-tetramer+ responses were gated on CD4+CD3+CD8-CD14-CD19-live lymphocytes. Gates identifying memory subsets (**C**-**D)**, chemokine receptors **(C)** and cytotoxic molecules **(D)** were defined on bulk CD4+ T cells, and then applied on M.tb-tetramer+ CD4+ T cells.

Supplementary Figure 2: Gene expression profiles of sorted M.tb-tetramer+ memory subsets.
(**A**) Principal component analysis (PCA) based on mRNA expression of the 20 differential expressed genes between M.tb-specific CD4 T cells subsets. (**B**) PC1 values of M.tb-specific CD4 T cell subsets. P-values were calculated with the Wilcoxon-matched pairs t-test, p-values < 0.025 were considered significant after correction for multiple comparisons (Bonferroni method).

Supplementary Figure 3: Detection of M.tb-tetramer+ cells in recent QFT converters.
(A) Frequencies of negative control and M.tb-tetramer responses detected in 19 adolescents (pooled time points). Median and interquartile range are indicated by solid black lines. Red dots denote tetramer+ staining that met criteria for further phenotyping (tetramer+ cells > 0.00214% of CD4+ T cells (dotted line) and 5-fold higher than negative control). Dots illustrate individual negative control- and M.tb-tetramer staining of 79 and 84 samples (19 participants with pooled time points), respectively. **(B)** Frequencies of CFP-10-tetramer+ (black) and ESAT-6-tetramer+ (blue) CD4+ T cells detected in each participant (n=12). P-values were calculated with the Wilcoxon-matched pairs t-test, p-values < 0.05 were considered significant and are shown.

Supplementary Figure 4: Detection of memory markers and subsets on M.tb-tetramer+ is affected by cryopreservation and/or Ab fluorochrome.
Median (error bars denote IQR) (A) expression of memory markers CD45RA, CCR7, CD27 and CD95 on M.tb-tetramer+ and (B) proportions of M.tb-specific T_{SCM} (CD45RA+CCR7+CD27+), T_{CM} (CD45RA-CCR7+CD27+) and T_{EFF} (CD45RA-CCR7-) detected in remotely M.tb infected adults using either fresh PBMC (n=28) or matched cryopreserved PBMC (n=20). P-values were calculated using Wilcoxon matched pairs t-test and p-values < 0.0125 and < 0.01667 were considered significant upon correction for multiple comparisons (Bonferroni method), for (A) and (B) respectively. C) Expression of CD95+ and CXCR3 on M.tb-specific T_{SCM} T cells. Symbols represents M.tb-specific T_{SCM} detected in recently M.tb infected adolescents (pooled time points) and remotely M.tb infected adults (fresh PBMC, n=28, and matched cryopreserved PBMC, n=20). PBMC stained fresh or

cryopreserved according staining protocol (see methods) are illustrated by grey (◇, T cell panel 2), teal (◇, T cell panel 3), or grey half-shaded symbols (—, T cell panel 4). P-values were calculated using Wilcoxon matched pairs t-test and p-values < 0.0167 were considered significant upon correction for multiple comparisons (Bonferroni method).

Supplementary Figure 5: chemokine receptor and cytotoxic molecule co-expression profiles of bulk and M.tb-tetramer+ memory subsets.
Box and whisker plots depicting proportions of (**A**) chemokine receptor (CXCR3, CCR6, CCR5 and CCR5) and (**B**) cytotoxic molecule (GrnA, GrnB, GrnK, granulysin and perforin) expressing M.tb-specific T_{SCM} (CD45RA+CCR7+CD27+, light blue), bulk CD4+ T_{CM} (CD45RA-CCR7+CD27+, n=28, magenta) and bulk CD4+ T_{EFF} (CD45RA-CCR7-CD27-, n=28, purple) CD4+ T cells. P-values were calculated using Wilcoxon signed-rank test and corrected for multiple comparison using the Benjamini Hochberg method with an FDR of 0.05. P-values < 0.05 were considered significant.
Box and whiskers plots depicting the proportion of M.tb-specific T_{SCM} (light blue), T_{CM} (magenta) and T_{EFF} (purple) CD4 T cells co-expressing (**A**) CCR4, CCR5, CCR6 and/or CXCR3 and (**B**) grnA, grnB, grnK, granulysin and/or perforin. P-values were calculated using Wilcoxon signed-rank test and Mann-Whitney test for chemokine receptor and cytotoxic molecules respectively, and corrected for multiple comparison using Benjamini Hochberg method with an FDR of 0.05. Adjusted p-values are shown. The number of participants meeting criteria for analysis of each M.tb-tetramer+ cell subset is as in Figure 4.

Supplementary Figure 6: M.tb-specific T_{SCM} produce Th1 cytokine in response to M.tb antigen stimulation.
Fresh whole blood from remotely M.tb infected (QFT+) adults (n=13) was left unstimulated or stimulated with peptide pools spanning M.tb antigens (Ag85B, ESAT-6, CFP-10 or BCG) for 12 hours. Cells from stimulated whole blood were gated according to the illustration. Antigen-specific cytokine responses were defined on CD4+CD3+CD8- lymphocytes, setting the gates on unstimulated cells. Gates identifying memory subsets, based on CD45RA and CCR7 expression, were set on bulk CD4+ T cells and applied to cytokine+ cells.

Supplementary Figure 7: M.tb-specific T_{SCM} can be defined using CD45RA and CCR7 staining only.
Comparison of protein expression (median fluorescence intensity, MFI) of CD27, CD95 and CXCR3 T_{SCM} markers between M.tb-specific T_{SCM} defined as CD45RA+CCR7+CD27+ (black) and CD45RA+CCR7+ (red, n=15 participants). P-values were calculated using Wilcoxon matched pairs t-test and p-values < 0.0167 were considered significant upon correction for multiple comparisons (Bonferroni method).

References

Gattinoni L, Lugli E, Ji Y, Pos Z, Paulos CM, Quigley MF, Almeida JR, Gostick E, Yu Z, Carpenito C, Wang E, Douek DC, Price DA, June CH, Marincola FM, Roederer M and Restifo NP (2011). A human memory T cell subset with stem cell-like properties. Nature Medicine 17, 1290–1297.
