# Supplementary figures and images for: Functional, Antigen-Specific Stem Cell Memory (TSCM) CD4+ T Cells Are Induced by Human Mycobacterium tuberculosis Infection
Source: Front Immunol. 2018 Mar 1;9:324. doi: 10.3389/fimmu.2018.00324 (PMC5839236; doi:10.3389/fimmu.2018.00324)

# Supplementary Figure 1

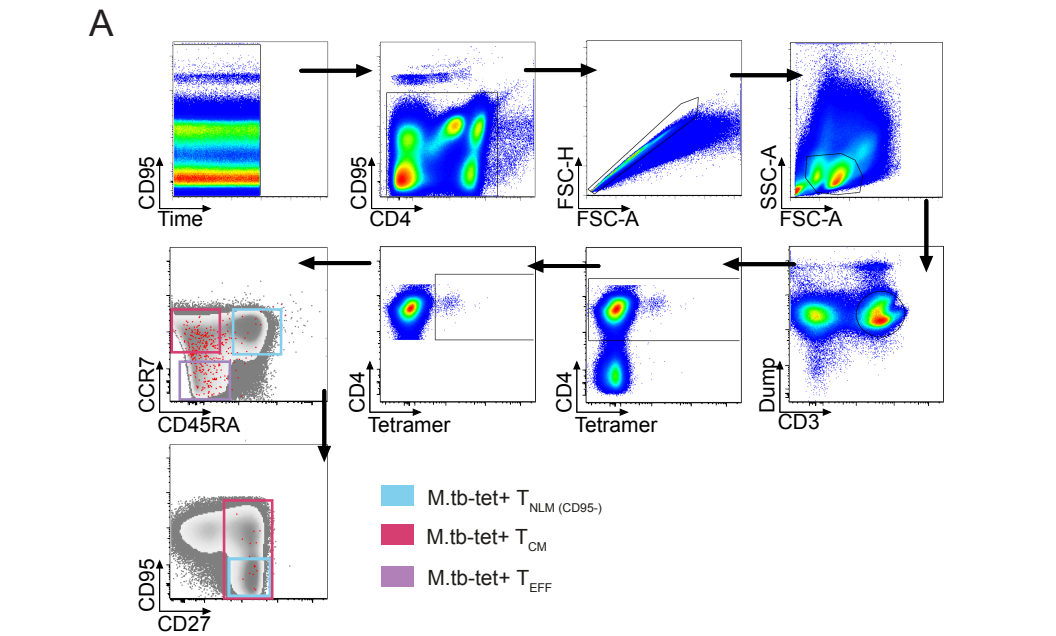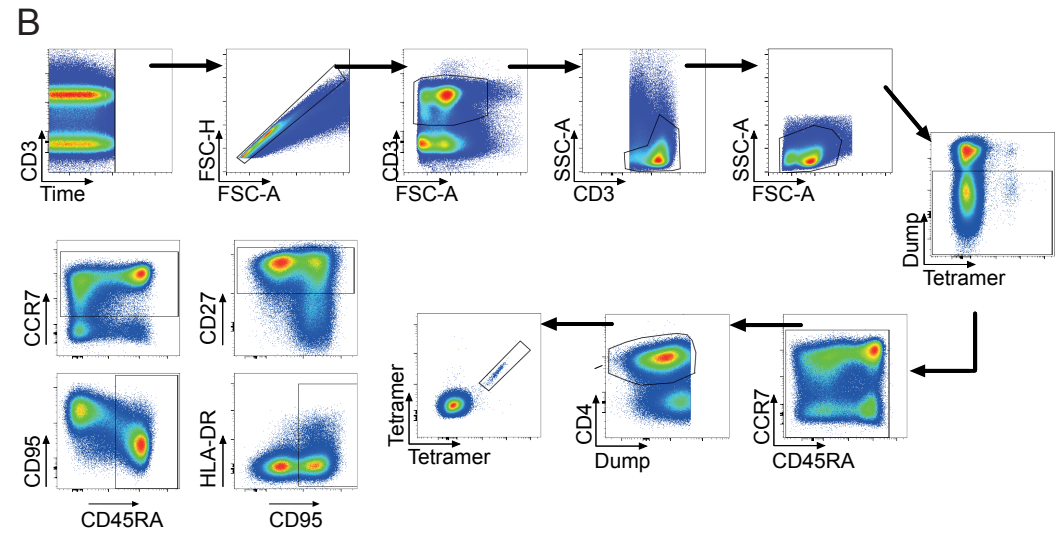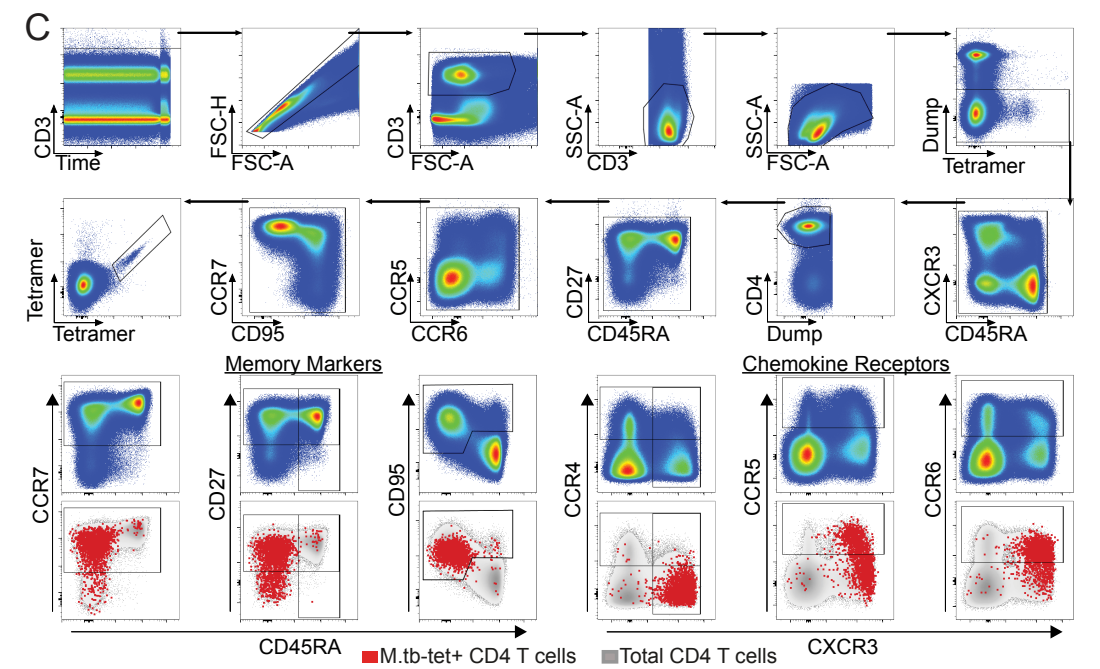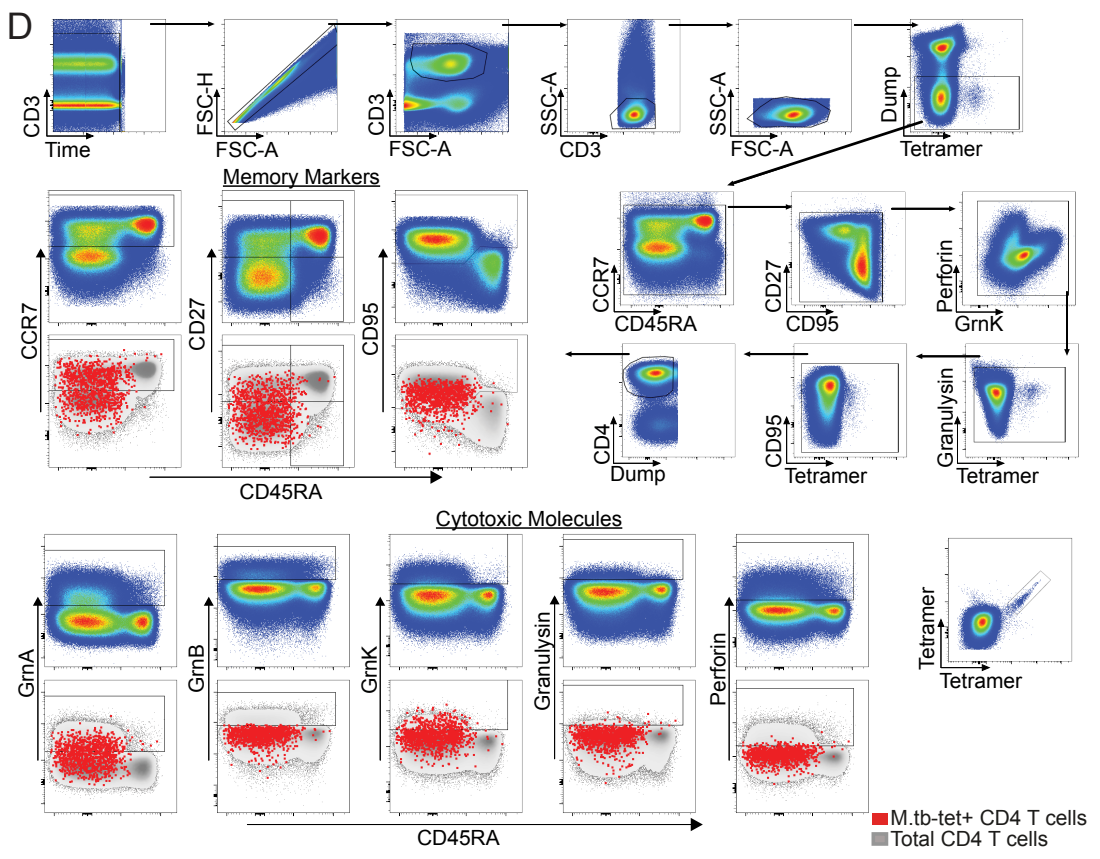

Supplement: Supplementary file 4 [file image_1.PDF]

## Supplementary Figure 2

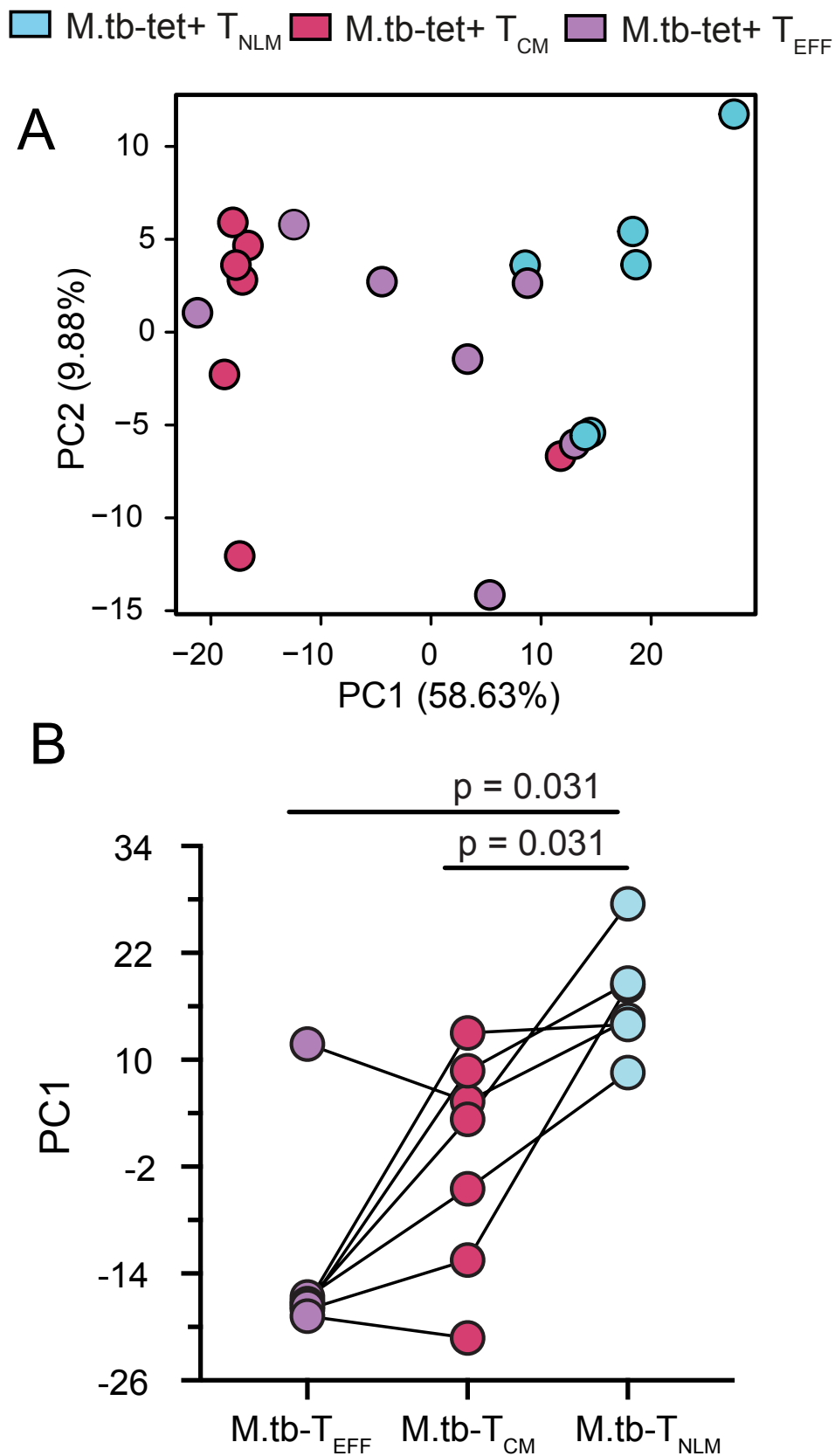

Supplement: Supplementary file 5 [file image_2.PDF]

Supplementary Figure 3

A

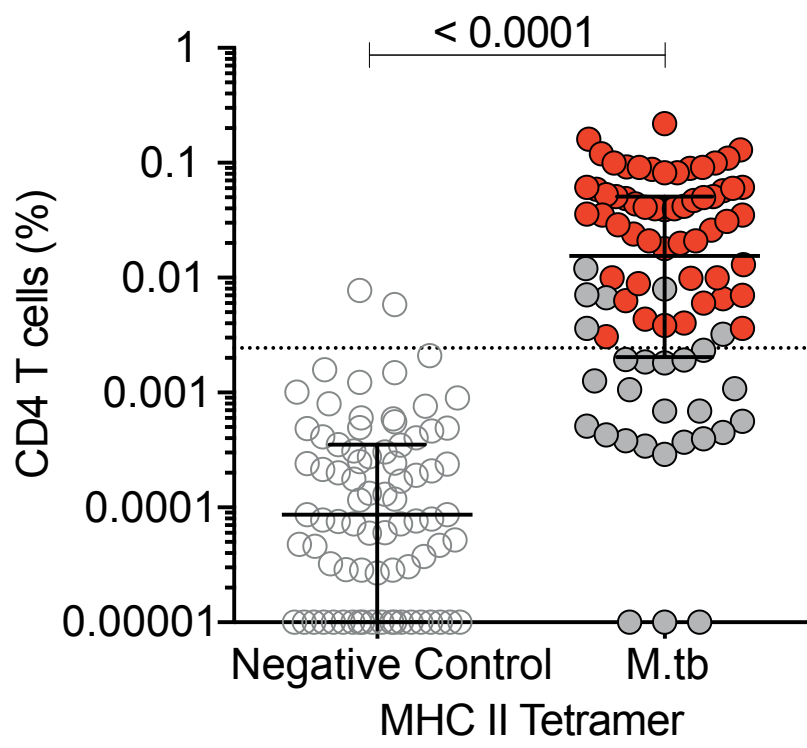

B

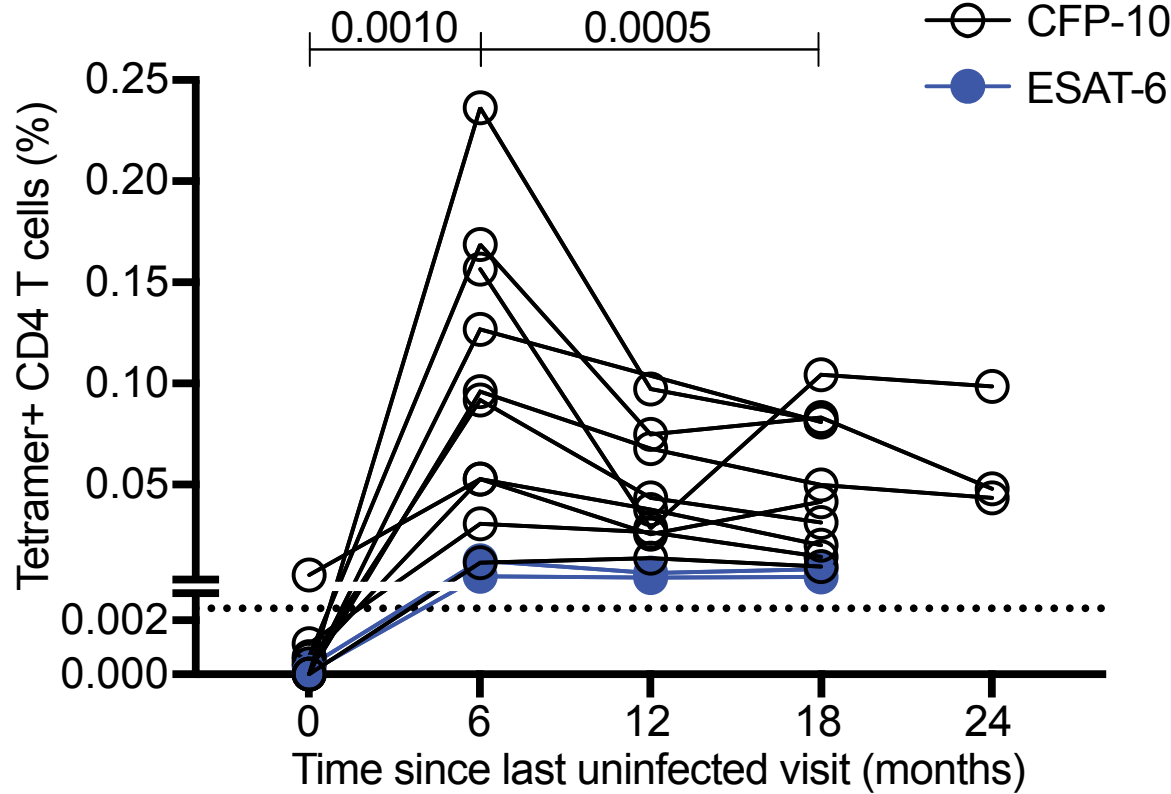

Supplement: Supplementary file 6 [file image_3.PDF]

Supplementary Figure 4

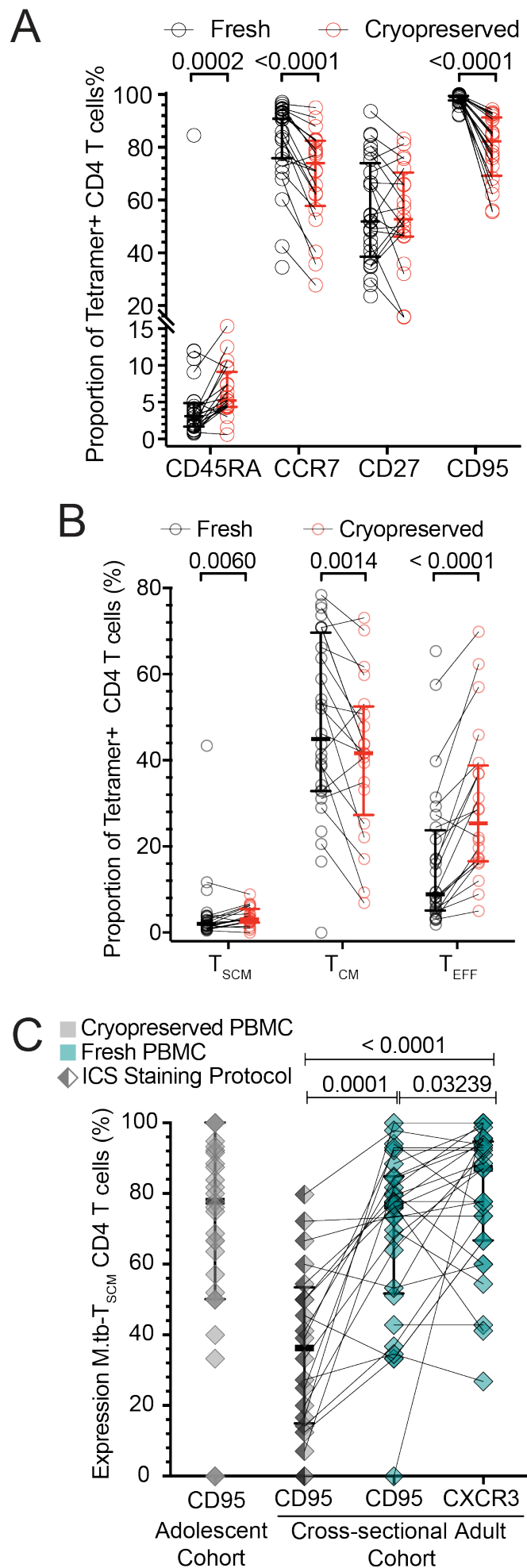

Supplement: Supplementary file 7 [file image_4.PDF]

Supplementary Figure 5

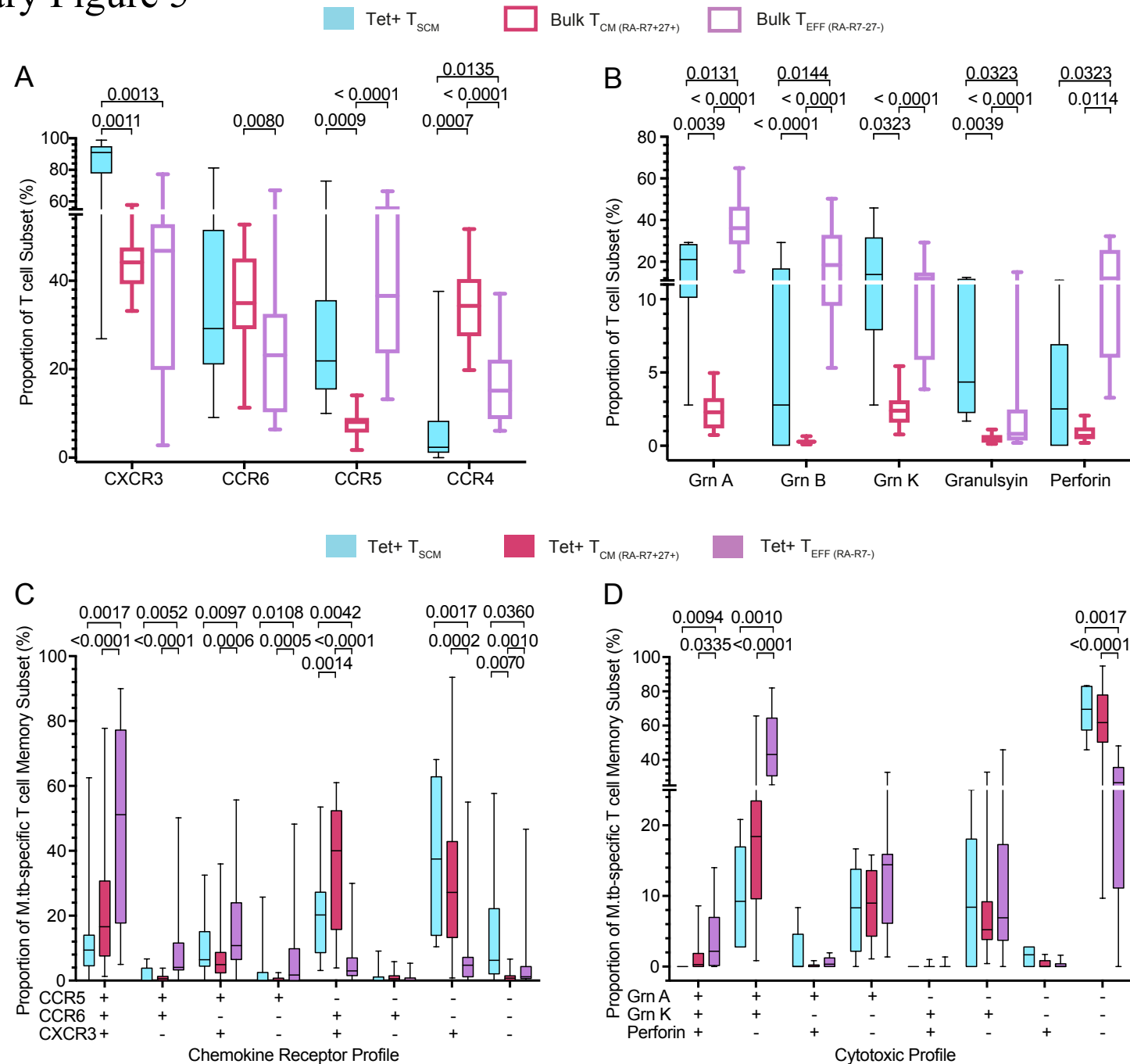

Supplement: Supplementary file 8 [file image_5.PDF]

Supplementary Figure 6

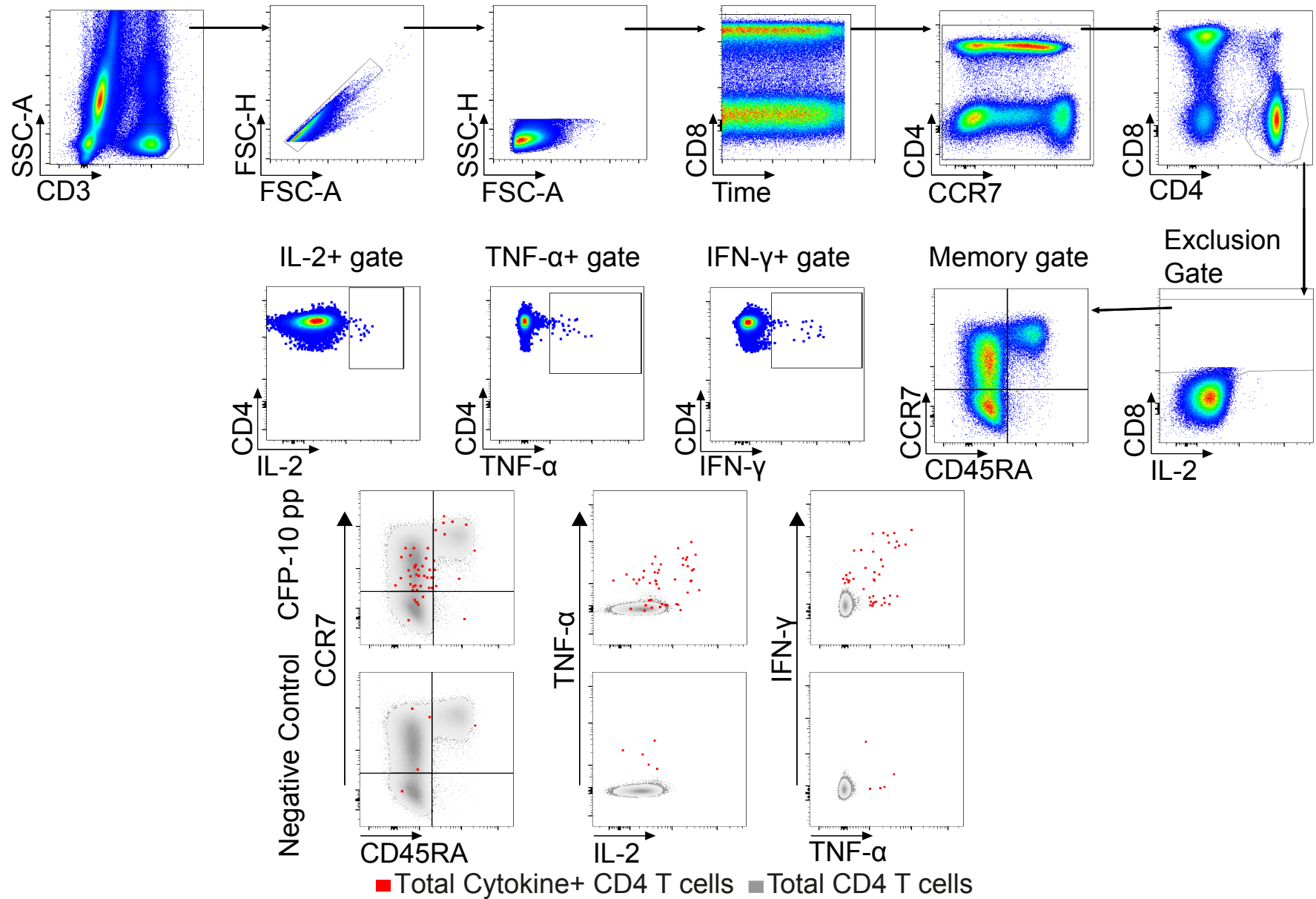

Supplement: Supplementary file 9 [file image_6.PDF]

Supplementary Figure 7

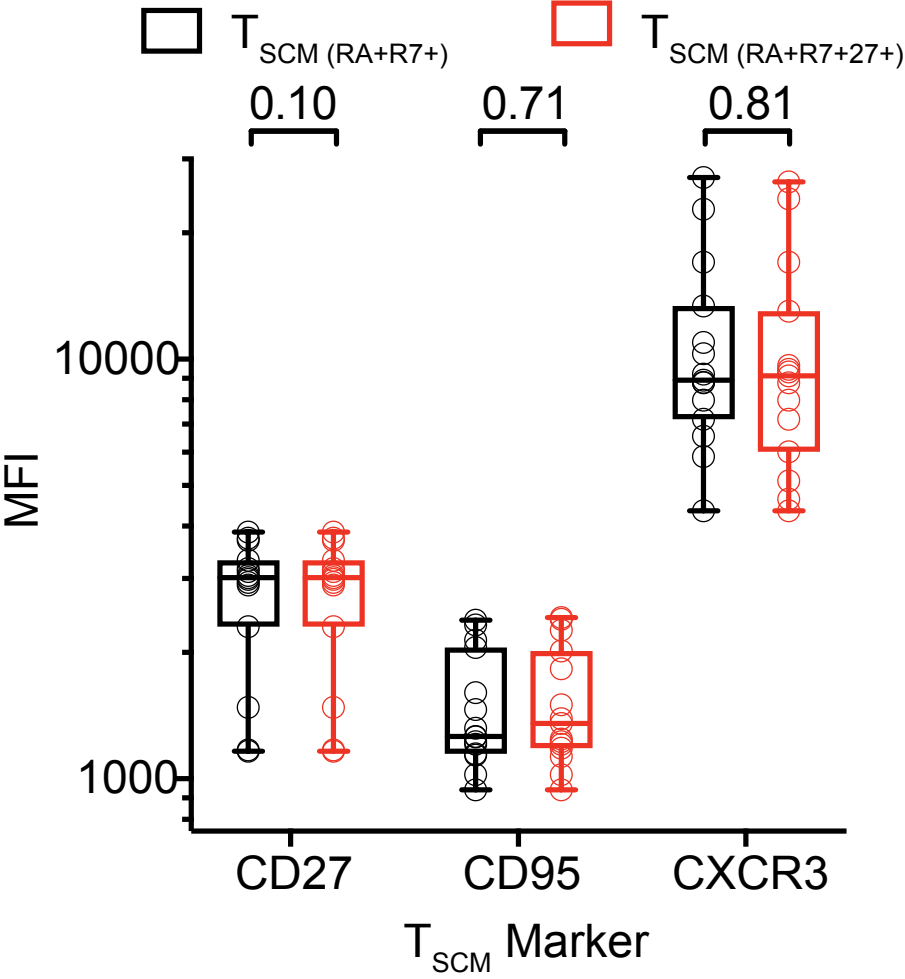

Supplement: Supplementary file 10 [file image_7.PDF]
